# Development of functional nanomedicines for tumor associated macrophages-focused cancer immunotherapy

**DOI:** 10.7150/thno.78572

**Published:** 2022-11-14

**Authors:** Xiao Wei, Jing Wang, Min Liang, Mingzhu Song

**Affiliations:** 1 School of Preclinical Medicine, Chengdu University, Chengdu 610106, P. R. China.; 2 Section of Molecular Dermatology, Medical Faculty Mannheim of Heidelberg University, Mannheim, Germany.; 3 Department of Thoracic and Cardiac Surgery, Affiliated Hospital of Chengdu University, Chengdu 610081, P. R. China.; 4 Evidence-Based Medicine Center, West China Hospital, Sichuan University, Chengdu, 610041, P. R. China.

**Keywords:** tumor associated macrophages, tumor immunosuppression, functional nanomedicines, cancer immunotherapy

## Abstract

Clinical cancer immunotherapies are usually impeded by tumor immunosuppression driven by tumor associated macrophages (TAMs). Thus, TAMs can be considered as a promising therapeutic target for improved immunotherapy, and TAMs-focused molecular targeting agents have made ideal progress in clinical practice. Even so, most TAMs-targeting agents still cannot cover up their own shortcomings as free drugs. The emergence of multifunctional nanomaterials can expectedly endow these therapeutic cargoes with high solubility, favorable pharmacokinetic distribution, cell-specific delivery, and controlled release. Here, the underlying mechanisms of tumor immunosuppression caused by TAMs are first emphatically elucidated, and then the basic design of TAMs-focused immune-nanomedicines are discussed, mainly including diverse categories of nanomaterials, targeted and stimulus-responsive modifications, and TAM imaging in nanomedicines. A summary of current TAMs-targeting immunotherapeutic mechanisms based on functional nanomedicines for TAMs elimination and/or repolarization is further presented. Lastly, some severe challenges related to functional nanomedicines for TAMs-focused cancer immunotherapy are proposed, and some feasible perspectives on clinical translation of TAMs-associated anticancer immunonanomedicines are provided. It is hoped that, with rapid development of nanomedicine in cancer immunotherapy, TAMs-focused therapeutic strategies may be anticipated to become an emerging immunotherapeutic modality for future clinical cancer treatment.

## Introduction

Immunotherapy is currently considered as a promising next-generation therapeutic strategy for various cancers thanks to its ability to modulate cell-specific immune responses toward tumors 1, which has gradually remodeled the landscape of clinical anticancer modality 2. In clinical practice, cancer immunotherapies have been well developed in recent years, mainly including cytokine therapy (e.g., IL-2 and IFN-γ), immune checkpoint blockade (ICB) therapy (e.g., anti-PD-1/PD-L1 antibodies), and adoptive T-cell transfer (e.g., chimeric antigen receptor (CAR) T-cell therapy) 3-5. Most of them generally follow the same pathway to make immune-activated cytotoxic T lymphocytes (CTLs) abundantly infiltrate into tumor tissues and destroy tumor cells 6. However, increasing data has indicated that the clinical potential of most immunotherapies is usually hampered by immunosuppressive status of the tumor microenvironment (TME) 7. Of note, the TME is rich in immunosuppressive cells (e.g., tumor-associated macrophages (TAMs), regulatory T cells (Tregs), immature dendritic cells (iDCs) and myeloid-derived suppressor cells (MDSCs)), that can suppress antitumor immunity as a result of secretion of various cytokines and chemokines, which enables tumor escape from immune surveillance 8.

Among most immunosuppressive cells, TAMs, as crucial drivers of immunosuppressive TME, account for the largest proportion of immune cells in the TME (approximately 50% of tumor mass) 9, 10, which possess highly heterogeneity and play a complex regulatory role in tumor immunity and immunotherapy due to helping tumor evade immune surveillance 11. Moreover, TAMs usually display M2-like phenotypes that exert tumor-promoting role in TME, and promote production of related immunosuppressive factors that trigger immunotherapy resistance, including cytokines, chemokines, growth factors and soluble signaling mediators 12. More importantly, accumulating studies have revealed that an abundant infiltration with M2-like TAMs is correlated with poor prognosis of patients in multiple cancers 13. Accordingly, TAMs can be deemed as a potential target for enhanced cancer immunotherapy by rebuilding the immunosuppressive TME. Numerous preclinical and clinical studies have also demonstrated the therapeutic potential of targeting TAMs to elevate the efficacy of cancer immunotherapies 11, 12, and TAMs-targeting drugs for diverse molecular signal targets, including chemical agents, nucleic acids, and proteins/peptides, have been well validated for effectively modulating antitumor immunity 14.

Although those therapeutic agents based on TAMs have achieved some expected progress in basic and even clinical studies, they still cannot cover up their shortcomings as free drug molecules, namely low solubility, fast metabolism, nonspecific tissue distribution, off-target toxicity and side effects, poor cellular uptake, etc. 15 Concurrently, another severe challenge, that impacts on the therapeutic effect of TAMs-based immune-medicines, largely originate from the complex immunosuppressive circumstance within tumors 16. Fortunately, with the rapid developments of nanotechnology in biomedicine, these typically intractable issues will be readily addressed to realize the enhancement of antitumor potency. By virtue of the unique superiority in nanoscale drug delivery platforms, the construction of novel versatile immune-nanomedicines can better help most immunomodulatory agents surmount those above hurdles, thereby selectively and effectively delivering to TAMs, and eventually reprogramming the immunosuppressive TME by modulating TAMs to promote adaptive immune responses to eradicate tumor cells 15. Besides, several types of nanomaterials, such as mesoporous silica (SiO_2_), iron oxide (Fe_3_O_4_) or polysaccharides, have been identified as promising immunoregulatory adjuvants that can directly provoke adaptive immune responses against tumors 17. Altogether, selecting and designing appropriate functional nanomedicines is essential to potentiate the antitumor immune efficacy by improving TAMs-driven immunosuppressive TME.

**Figure 1:**
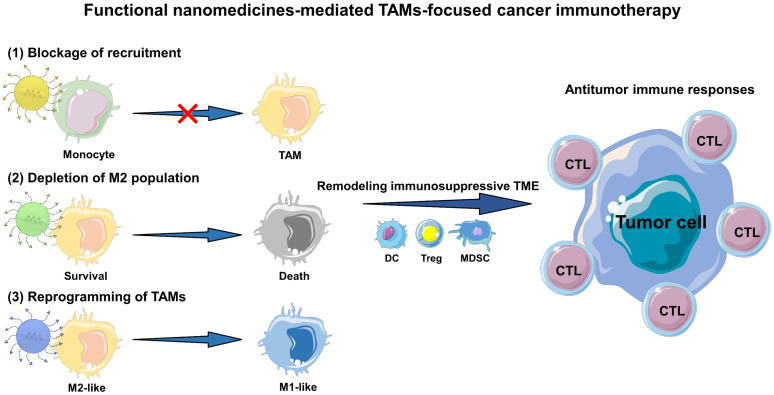
Schematic illustration of functional nanomedicines-mediated TAMs-focused cancer immunotherapy.

In this review, we outline the current advances of functional nanomedicines mediated strategies in TAMs-focused cancer immunotherapy (**Figure 1**). Other studies in TAMs-targeting therapeutic strategies without the involvement of immune are not included. Collectively, there are three main types of TAMs-centered immunotherapies, including (1) blockage of bone-marrow-derived TAMs recruitment, (2) depletion of tumor-resident TAMs population, and (3) reprogramming of TAMs phenotype. We first detailedly explain the molecular mechanisms by which TAMs induce tumor immunosuppression, and then focus on the rational design of TAMs-centered functional nanomedicines, which involves multiple categories of nanomaterials with different physicochemical characteristics, and diverse functional modifications including targeting capacity, stimulus-responsive function and TAM imaging. Furthermore, we present an overview of current functional nanomedicines for different TAMs-targeting immunotherapeutic mechanisms. Finally, we mainly discuss some severe challenges in this research field, and provide some feasible perspectives on nanomedicines mediated TAMs regulation to alleviate the tumoral immunosuppressive microenvironment for effective cancer immunotherapy.

## Mechanisms of TAMs-driven tumor immunosuppression

In general, TAMs in TME are largely constituted by myeloid-derived and tissue-resident macrophages, both of which can elicit the undesirable tumor immunosuppression 12. TAMs usually present different phenotypes, which can be polarized into antitumour M1-like macrophages and protumoral M2-like macrophages due to their plasticity and diversity 12. Of which, M1-like macrophages display tumoricidal effect by the secretion of abundant cytokines such as interleukin-12 (IL-12) and chemokines (e.g., chemokine (C-X-C motif) ligand 9/10 (CXCL9/10)), that can dominate T cell-mediated antitumor immune response 18-20. Of note, infiltration of M1-type macrophages is an independent prognostic factor for overall survival in several cancers 21-23. However, in the TME of most solid tumors, TAMs mainly tend to polarize into immunosuppressive M2-like phenotypes thanks to releasing soluble factors such as interleukin-6 (IL-6) and prostaglandin E_2_ (PGE_2_) by tumor cells 24-26, which may play a prominent role in promoting the tumor progression by inhibiting antitumor immune response 26. Indeed, increasing evidence has indicated that TAMs can be regarded as immunosuppressive regulators in tumor immunity and immunotherapy 27. Thus, understanding the diversity and plasticity of TAMs and their regulatory mechanisms within the complex TME are vital to inspire the development of appealing immunotherapeutic modalities with increased antitumor efficacy and decreased side effects. Herein, two major mechanisms involved in the interactions of TAMs with immune cells and tumor cells for the development of tumor immunosuppression are described in detail.

### The interactions of TAMs and immune cells

**Figure 2:**
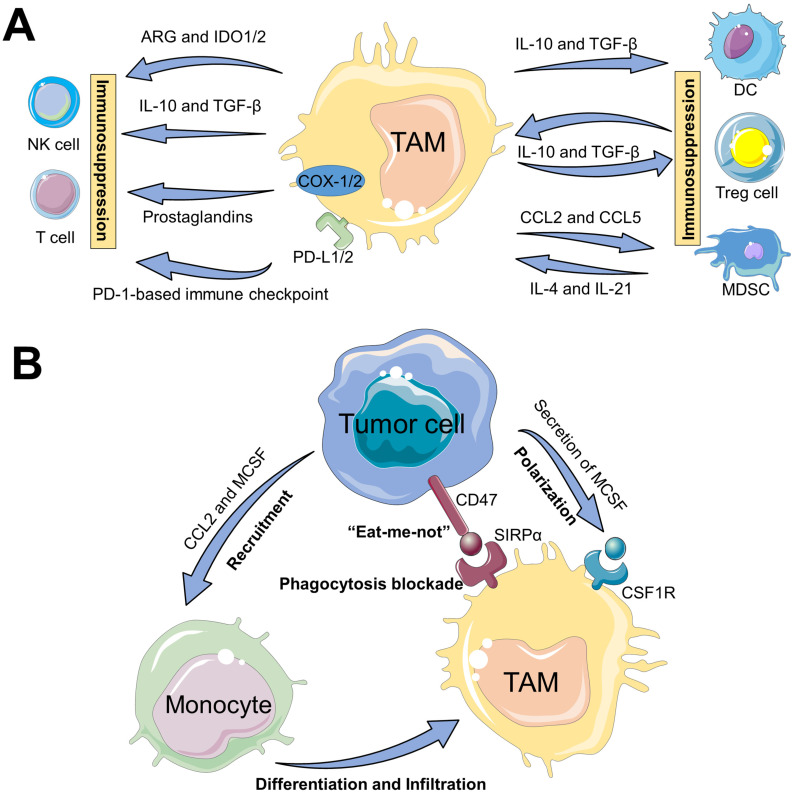
The underlying mechanisms of TAMs-driven tumor immunosuppression, including** (A)** the interactions of TAMs and immune cells and** (B)** TAMs-mediated immune escape of tumor cells.

The underlying mechanisms of tumor immune suppression driven by TAMs may be classified into two pathways (**Figure 2A**). First, TAMs can directly weaken the activities of T cells and NK cells, mainly including (i) TAMs often release IL-10 and TGF-β cytokines to suppress the immune function of T cells or NK cells 11; (ii) TAMs contribute to amino acid metabolic starvation of T cells or NK cells due to the activity of arginase and/or production of immunosuppressive metabolites through the indoleamine-pyrrole 2,3-dioxygenase 1/2 (IDO1/2) pathway 11, 28; (iii) cyclooxygenase 1/2 (COX-1/2) expressed in TAMs promote the production of prostaglandins via arachidonic acid metabolism, which leads to immunosuppressive effects on T cells 29; (iiii) TAMs express cell surface proteins such as programmed death ligand 1/2 (PD-L1/2) that induce the inhibitory PD-1-mediated immune checkpoint in T cells or NK cells 11, 30. Second, TAMs can indirectly restrain the immune functions of T cells via impact on other immune cells such as Tregs, DCs or MDSCs. In detail, TAMs can promote the immunosuppressive activity of Tregs through bidirectional interactions and block DCs maturation, both of which are driven by TAM-produced immunosuppressive cytokines (e.g., IL-10 and TGF-β), thereby impeding CTLs-triggered specific antitumor responses 31, 32. Moreover, MDSCs may differentiate into M2-like TAMs within TME through a series of cytokine stimulation (e.g., IL-4 and IL-21), and TAMs release chemokines such as chemokine (C-C motif) ligand 2/5 (CCL2/5) to recruit MDSCs infiltration into tumors 30, thereby resulting in the aggravation of the immunosuppression state. Taken together, these immunosuppressive activities presented in TAMs block the specific antitumor immune responses owning to their expression of cell surface receptors, secreted cytokines, chemokines, and enzymes that modulate the functions of several immune cells.

### TAMs-mediated immune escape of tumor cells

In addition, tumor cells can directly evade the innate immune system through the crosstalk with TAMs (**Figure 2B**). One such major mechanism is that, tumor-secreted macrophage colony stimulating factor (MCSF) can bind to colony stimulating factor 1 receptor (CSF1R) on TAMs, thus initiating the MCSF-CSF1R signaling pathway to recruit and polarize TAMs into immunosuppressive M2-like phenotypes 19, 33. The other molecular mechanism involved in the CD47-signal regulatory protein α (SIRPα) pathway is namely that, engagement of SIRPα expressed on TAMs with CD47 overexpressed on tumor cell surface can activate the Src homology region 2 domain-phosphatases 1/2 (SHP-1/2) in TAMs, thereby resulting in a “eat-me-not” signal and inhibition of macrophage-phagocytosis 34. Furthermore, tumor-derived chemokines and cytokines (e.g., CCL2 and MCSF) are also the pivotal stimulating factors that promote the recruitment of circulating myeloid-derived monocytes 25, which contributes to the infiltration of TAMs into tumor regions.

## Rational design of TAMs-focused functional nanomedicines

The molecular signaling mechanisms described above have clearly proved the fact that TAMs can trigger immunosuppression of the TME, which will facilitate the clinical development of a wide variety of TAMs-targeting agents that augment the anticancer therapeutic efficacy via remodeling the immunosuppressive microenvironment 12. However, as described in “Introduction”, there are many severe issues that impede antitumor efficacy of TAMs-targeting agents, so we need to use the burgeoning nanotechnology to arm these molecular targeted drugs and ultimately boost TAMs-targeting immunotherapy.

Even so, there are currently some challenges in delivering drugs to TAMs with nanoplatforms. First, nanoscale size is identified as a common physical feature in nanocarriers. Relatively large nanoparticles (NPs) can be designed to avoid invading capillaries, while small enough NPs can be used to avoid phagocytosis by the reticuloendothelial system (RES) 35. Second, the morphology and charge of NPs also affects the safety, pharmacokinetic properties and bioavailability of the cargoes, while determining the biodistribution and cell internalization 35. Third, although the enhanced permeability and retention (EPR) effect of tumor vessels may promote the tumor accumulation of nanocarriers, the complex TME may hinder the uptake of TAMs. Therefore, NPs modified with different TAMs targeting molecules can enhance the corresponding cellular specificity and uptake. Last, to timely and effectively release drugs into the TAMs, stimulus-responsive NPs can be fabricated to trigger the release of the cargoes to specific sites under different external or internal stimuli, thereby increasing the concentration of drug in tumor sites 35. Overall, the principles of design need to consider appropriate nano-structure/morphology, uniform particle size, reasonable zeta potential, TAMs-targeting capacity, and stimulus response under physiological conditions within TAMs, which will be extremely beneficial to precisely deliver drugs into TAMs. In addition, accumulating data has also proved that drug-loaded NPs are largely uptook by TAMs within tumor tissues while they aim to delivering drugs to the tumor cells 36-38, which significantly weakens the ability of NPs to eradicate tumor cells. Therefore, the engulfment of NPs by TAMs is usually regarded as a biological obstacle for NPs-mediated tumor-targeting therapy, but conversely, TAMs should become a potential therapeutic target because of their ability to actively hijack tumor-targeted nanomaterials in TME. Based on this characteristic, a series of ideal multifunctional nanoplatforms can be reasonably designed for the targeted delivery of immunomodulatory drugs into M2-like TAMs, thereby regulating TAMs to activate the antitumor immune response. Indeed, TAMs-focused nanotherapeutic strategies have the potential to synergize with improved anticancer immunotherapy 30.

The following is the main discussions of many types of NPs with different physicochemical properties and multifunctional modifications of nanoplatforms for TAMs-focused tumor immunotherapy. Based on recent studies in this field, various TAMs-focused functional nanomedicines for cancer immunotherapy are also summarized in **Table 1**.

### Various nanomaterials for TAMs-focused immunotherapy

**Figure 3:**
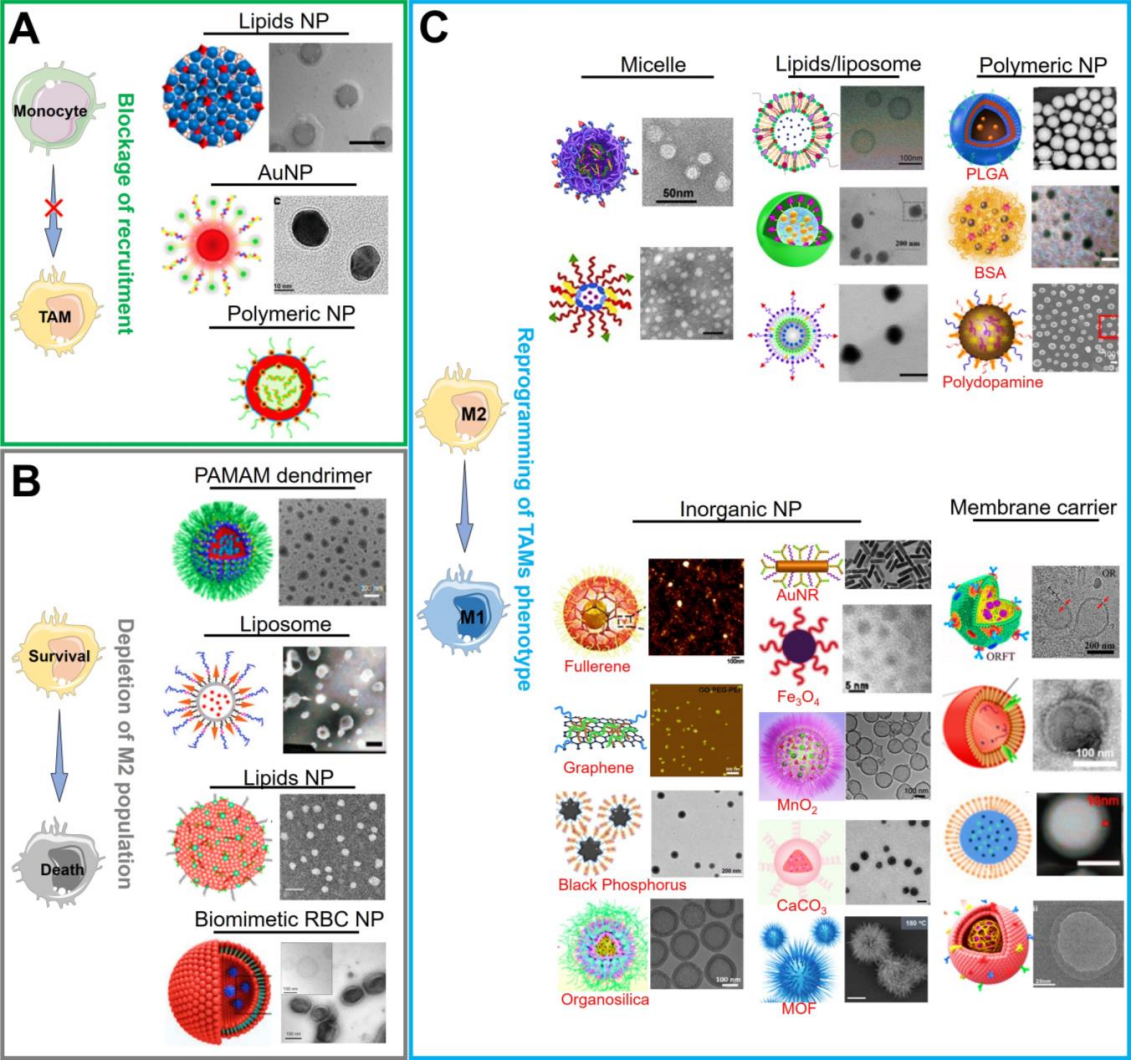
Current functional nanomedicines for different TAMs-focused cancer immunotherapeutic strategies, including **(A)** blockage of recruitment, **(B)** depletion of M2 population, **(C)** reprogramming of TAMs phenotype. Partial images were adapted with permission from 42, copyright 2019 Royal Society of Chemistry; 46 copyright 2020, 47 copyright 2020, 60 copyright 2014, 62 copyright 2021, 64 copyright 2019, 65 copyright 2014, 77 copyright 2019, 78 copyright 2020 Elsevier Ltd.; 41 copyright 2017, 50 copyright 2018, 59 copyright 2020, 61 copyright 2020, 71 copyright 2019, 72 copyright 2018, 74 copyright 2018, 79 copyright 2018, 80 copyright 2017 American Chemical Society; 44 copyright 2019, 66 copyright 2015, 81 copyright 2021, 82 copyright 2019, 83 copyright 2019, 84 copyright 2020, 85 copyright 2020 WILEY-VCH Verlag GmbH & Co. KGaA, Weinheim; 51, copyright 2021 Ivyspring International Publisher; 67 copyright 2017, 86 copyright 2019 Springer Nature Limited.

There are currently a variety of NPs based on different material categories, including organic nanomaterials (e.g., lipids/liposomes, polymeric micelles and polymeric NPs), inorganic nanomaterials (e.g., carbon-based NPs, silicon-based NPs, and metals-based NPs (gold, manganese, zinc and iron)), and biological/natural carrier-based nanomaterials, which can be considered for the effective load and delivery of drugs in TAMs-centered cancer immunotherapy (**Figure 3**). As mentioned above, NPs are more readily internalized by TAMs during their delivery of targeted drugs to tumor cells, implying that they are latent drug carriers for macrophages. Furthermore, their physical characteristics such as size, charge or morphology may impact the phagocytosis effect of TAMs while NPs are constructed to target and modulate TAMs 8, 76. Overall, the appropriate selection of carrier materials is a prerequisite for effective TAMs-targeting immunotherapy. Next, we emphatically introduce the progress on the organic, inorganic and biomimetic nanomaterials in TAMs-focused immunotherapy.

### Organic nanomaterials

#### Lipids/liposomes

Lipids-based NPs or liposomes are often used as ideal drug delivery systems in cancer treatment, which possess the ability to encapsulate diverse therapeutic agents such as lipophilic drugs, hydrophilic drugs, or nucleic acid drugs (e.g., small interfering RNA (siRNA)) responsible for their natural phospholipid bilayer structure 87. In view of drug loading ways, these NPs can either encapsulate hydrophobic drugs into the lipid bilayer through hydrophobic forces, or import hydrophilic drugs in inner core through passive diffusion, even electrostatically adsorb nucleic acid drugs on the surface of cationic liposomes 87. For example, Ramesh et al. designed a liposome-based immunotheranostic nanoreporter system by concurrently incorporating a CSF1R inhibiting (iCSF1R) amphiphile along with a nitric oxide (NO) fluorescence probe 39, in which both hydrophobic iCSF1R and NO probe occupied the spaces between the lipid bilayer. These liposomal NPs with a mean size of around 109 nm were mainly formed by self-assembly of phosphatidylcholine (PC) and 1,2-distearoyl-sn-glycero-3-phosphoethanolamine-N-(amino(polyethyleneglycol)-2000) (DSPE-PEG2000), which not only achieved the augmented antitumor efficacy of breast cancer through sustained inhibition of CSF1-CSF1R signaling axis, but also enabled real-time monitoring of TAMs-centered immunotherapy response that relied on immunomodulation of TAMs from a protumoral M2-subtype to an antitumoral M1-subtype. Sousa et al. indicated that the soluble factors (e.g., enzymes, cytokines and growth factors) secreted from breast cancer cells could modulate TAMs toward immunosuppressive M2 phenotype 40. To solve it, liposomal nanomedicines that physically wrapped bisphosphonates (e.g., clodronate and zoledronate) were developed to counteract this modulation. As a result, zoledronate-loaded liposomes exerted antitumor immune responses by re-educating M2-type TAMs in breast cancer 88, and clodronate-loaded liposomes may be inclined to induce apoptosis upon cell internalization for TAMs depletion 88. In a research by Qian et al. 41, they built a sub-30 nm lipid NP that largely consisted of 1,2-dimyristoyl-sn-glycero-3-phosphocholine (DMPC), DSPE-PEG2000 and cholesterol oleate for efficient penetration in melanoma tumors, in which an anti-CSF1R siRNA (siCD115) was loaded on their surface by inserting into the lipid monolayer. Then the lipid NPs would be expected to effectively deliver siCD115 to TAMs for the specific blockade of their survival signal (CSF1-CSF1R), resulting in the direct elimination of M2-like TAMs from melanoma and the activation of CD8^+^ T cells-mediated antitumor responses. Besides, several nanoformulations based on liposomal NPs have achieved some anticipated advance in clinical trials or on the market 88, implying that lipids/liposome-mediated TAMs-centered antitumor strategies are highly worthy of development.

#### Polymeric micelles

Among most polymers, amphiphilic polymers, that can self-assemble into core-shell micellar NPs in aqueous solution, are mostly used as nanocarrier materials for the drug delivery in cancer therapy, which is beneficial to increase solubility and bioavailability of therapeutic agents 87. Poly(ethylene glycol) (PEG) is commonly employed as the micellar hydrophilic segments because of its ability to prolong the half-life of drugs 87. The inner core of micelles consisting of hydrophobic polymers (e.g., poly(ε-caprolactone) (PCL) and poly(lactide) (PLA)) is generally served as a container for hydrophobic drugs 47. In order to carry anionic nucleic acid drugs (e.g., plasmid gene and siRNA), the hydrophilic segment of the micelles can be replaced by cationic polymers (e.g., polyethyleneimin (PEI)) 46. On the basis of these principles, polymeric micelles can become an appealing nanocarrier for the effective delivery of TAMs-targeting immunoagents with different physicochemical characteristics. For instance, Li et al. used a mixed nanomicelle formulated with PEI-stearic acid and DSPE-PEG2000 to co-load a PI3K-γ inhibitor (BEZ235) via hydrophobic forces and an anti-CSF1R siRNA through electrostatic adherence for remodeling immunosuppressive TME caused by M2-type TAMs in pancreatic cancer 46. The size and zeta potential of these nanomedicines displayed nearly 79 nm and 5 mv, along with the 82.2% encapsulation efficiency of BEZ235 and 85% transfection efficiency of siRNA. As was expected, NPs-assisted dual blocking agents were effectively transported to the pancreatic tumor tissues and targeted TAMs. For another example, Kudo et al. were inspired by the production of NO in M1-like TAMs at an early stage of cancer responsible for the overexpressed inducible NO synthase (iNOS) catalyzing the substrate L-arginine (L-Arg) 48, 89, which could direct trigger apoptosis of tumor cells. So, a PEG-P(L-Arg) copolymers and chondroitin sulfate (CS) were designed to form polyion mixed micelles. Of which, P(L-Arg) was not only the hydrophobic moiety of the micellar system, but also the precursor of NO production. Indeed, this micelle-assisted arginine delivery system presented promise as a TAMs-focused immunotherapeutic approach through NO-mediated cytotoxity of colorectal cancer. In addition to conventional amphiphilic polymers as micellar platforms, some polymers (e.g. poly(amidoamine) (PAMAM) dendrimer) themselves also have the potential to target and activate TAMs 49, 90, which well favors the development of ideal nanocarriers for selective delivery of immunomodulatory reagents to TAMs and further augment antitumor immunotherapy.

#### Polymeric NPs

Polymeric NPs also hold great promise as drug delivery systems for improved cancer immunotherapy by immunomodulating TAMs and directing the changeover of immunosuppressive TME, which can encapsulate drug molecules through hydrophobic or electrostatic interactions along with a high drug-loading efficiency 87, 91. Else, these NPs are featured with ease of surface functionalization for targeted and responsive drug delivery. For some polymers such as poly(lactic-co-glycolic acid) (PLGA) 50, poly(β-amino ester)s 92 and polydopamine (PDA) 56, they possess the ability to form the proper nanocarriers for the targeted delivery of TAMs-focused immunotherapeutics. Shi et al. used PEGylated PLGA NPs to physically incorporate photosensitizers indocyanine green (ICG) and titanium dioxide (TiO_2_) for initiating reactive oxygen species (ROS) photogeneration in endosome/lysosome or cytoplasm of TAMs 50, mainly aiming to reprogram TAMs toward an immune-activated M1 phenotype for tumor antigen presentation and CTLs recruitment 93, 94. Dual-photosensitizers loaded PLGA NPs displayed well-defined spherical shape, uniform size (60-95 nm), low zeta potential (nearly -1.5 mv) and controllable loading capacity with declined phototoxicity, which laid a stable foundation for subsequent antitumor immunotherapy via photodynamic immuomodulation of M2-like macrophages. Besides, another polymer like PEI can also be used as the major components of polymeric NPs that specifically deliver TAM-targeting agents. For instance, Parayath et al. used HA-PEI NPs encapsulating miR-125b for TAM-specific delivery and transfection in lung cancer immunotherapy, which showed a proper diameter of 92 nm and could facilitated electrostatic interaction between positively charged PEI and negatively charged miRNA 58.

Several types of biomacromolecules like proteins (e.g., bovine serum albumin (BSA) and human ferritin) 83, 95 or polysaccharides (e.g., CS, dextran and alginate) 96, 97 can be used as drug vehicles for TAMs-based tumor immunotherapy. For protein-based NPs, Yu et al. constructed a versatile immune-nanoregulator based on BSA as a drug carrier to simultaneously encapsulate MnO_2_ particles and small molecular IPI549, which remodeled TAMs-mediated immunosuppressive TME and liberated the immune system against tumors 83. They verified that the BSA NPs showed spherical shape with uniform size distribution (about 65 nm), and drug-loaded NPs were stable in a simulated blood environment. Also, ferritin with a good biocompatibility can be utilized as a natural drug delivery vector, which disassembles under a strong acidic pH environment and reassembles once pH value restores to the neutral condition 98. Terashima et al. have validated that human ferritin hold the ability to intrinsically target macrophages 99. Inspired by the prior knowledge, Shan et al. constructed a human ferritin-based nanoplatform for the targeted delivery of a Toll-like receptor 9 (TLR9) agonist (CpG oligodeoxynucleotide (ODN)) to M2-like TAMs in breast cancer, resulting in the repolarization of TAMs to tumoricidal M1 phenotype that elicited specifically immunoreaction 95. The ferritin NPs presented homogenous spherical cage-like structures along with an average diameter of approximately 20 nm and a zeta potential of about -12 mv, which facilitated the elusions of some *in vivo* delivery barriers and the tumor penetration of NPs after intravenous injection. For polysaccharide NPs, Huang et al. employed PEG-histamine-modified alginate (PHA) to combine the galactosylated cationic dextran loaded with three types of ODNs (CpG, anti-IL-10 and anti-IL-10RA) to form a nano-complex, which directed the reversion of TAMs phenotype and activated their antitumor immunity 97.

Besides, poly(amino acid) can be considered as a latent carrier framework. Liu et al. fabricated mixed polypeptide NPs based on n-butylamine-poly(L-lysine)-b-poly(L-cysteine) (PLL-PLC) polypeptides coated with sheddable PEG-PLL 55, to effectively facilitate TAMs-targeted microRNA-155 (miR155) delivery for repolarizing M2-type TAMs against tumors, in which the cationic PLL loaded miR155 via electrostatic forces, and the thiol groups of PLC drove themselves self-crosslinking to form a structural stable nanocarrier. Moreover, these hybrid polypeptide NPs possessed the charge-reversible property that presented the zeta potential changes from +35 mv to +5 mv for the protection of NPs and TAMs-targeted miR delivery through escape from *in vivo* delivery barriers, along with an ideal particle size of almost 100 nm that was a key determinant of physiological stability during blood circulation 100. For another poly(amino acid), poly(glutamic acid) (PGA) has been adopted by Castro et al. to form pro-inflammatory NPs 101. To be specific, a chitosan-PGA NPs with a uniform size of 183 nm and a positive charge of 18.7 mv can successfully activate immunostimulatory macrophages and DCs, thus promoting an obvious increase of effector T cells.

### Inorganic nanomaterials

Inorganic materials, including carbon (C), silicon (Si), phosphorus (P), metal, etc., also have the tremendous potential as drug delivery NPs on account of their small sizes, large surface areas, ease of surface modifications, and facile synthesis, which have attracted extensive attention for TAMs-focused cancer immunotherapy.

#### C-based nanomaterials

In terms of C-based nanomaterials, fullerenol (C_82_(OH)_22_) and fullerene (C_82_) have been well validated as latent immunoregulatory NPs to target TAMs for priming T cell-mediated anticancer immunotherapy by reversing the immunosuppressive TME 59, 102. Additionally, graphene oxide (GO), as a graphene derivative, holds promise as robust nanocarrier for the delivery of immunoregulatory drugs to M2-type TAMs, and graphene itself can be used as an available immunoadjuvant to effectively stimulate the maturation of macrophages with the release of proinflammatory cytokines 103, 104. For instance, Tao et al. employed GO functionalized with PEG and PEI as promising nucleic acids vectors to incorporate immunoadjuvant CpG via electrostatic binding for synergistic photothermal-immunotherapy, thus efficiently boosting the immune responses toward CT26 colon carcinoma through TLR9-induced TAMs activation 60. Of which, GO-PEG-PEI showed a dispersed piece-like structure with a thickness of about 10 nm, along with the zeta potential of nearly +38 mV thanks to covalent conjugation of the cationic PEI that dominated the loading of the CpG.

#### Si-based nanomaterials

As another inorganic nanomaterial, mesoporous SiO_2_ are commonly regarded as a promising drug delivery platform because of their intrinsic biocompatibility, various functionalization and well-defined porous structures in cancer therapy 105, which can directly target and modulate the immunosuppressive phenotype of TAMs, thus priming the specific immune responses against tumors 106. Li et al. engineered hollow mesoporous organosilica NPs (HMONs) as a reservoir of chemotherapeutic 10-hydroxycamptothecin (HCPT) and the monocarboxylate transporter (MCT) siRNA, both of which were separately encapsulated into this nanoplatform through hydrophobic and electrostatic forces 61. HMONs owned a mean size of about 180 nm with an initial mesopore diameter of 3.886 nm, and the surface areas of 634.59 m^2^/g with corresponding pore volumes of 1.275 cm^3^/g, which played a pivotal role in the subsequent efficient loading of HCPT and siRNA. As expected, these nanomedicines lastly not only triggered tumor cell apoptosis, but also drove the polarization of TAMs and the recovery of CTLs activity for synergistic chemo-immunotherapy in both B16F10 melanoma and 4T1 breast cancer.

#### P-based nanomaterials

Black phosphorus (BP), as a typical two-dimensional layered inorganic material, has been served as desirable antitumor NPs responsible for its biosecurity and photothermal conversion capacity 107, 108. Zhang et al. developed BP NPs conjugated with low molecular weight hyaluronic acid (HA) (MW < 5 kDa) that can be applied for concurrent tumor-targeting and TAMs-reprogramming for breast cancer photo-immunotherapy 62. HA-BP NPs displayed an average diameter of around 56 nm with a zeta potential of -28.4 mv, implying that they could maintain stability and promote tumor accumulation during* in vivo* delivery.

#### Metal-based nanomaterials

Metal-based nanomaterials, like gold nanorods/NPs (AuNRs/AuNPs) 65, 66, 109, Fe_3_O_4_ NPs 64, manganese dioxide (MnO_2_) NPs 67, zinc oxide (ZnO) NPs 110, and calcium carbonate (CaCO_3_) NPs 86, have been well verified as impressive immune-activated platforms for TAMs-associated antitumor immune responses. Among them, Fe_3_O_4_ NPs and ZnO NPs have become the U.S. Food and Drug Administration (FDA)-approved nanoproducts owning to their structure stability, biocompatibility and biosafety 111, 112. In particular, it has been firstly reported that Fe_3_O_4_ NPs can stimulate and reverse TAMs phenotype from immunosuppressive M2 type to immune-activated M1 type 113, thereby leading to the activation of specific antitumor immune response. Besides, metal-organic framework (MOF), as a class of metal-based hybrid materials, has been deemed as a promising next-generation drug delivery platform, featured with composition versatility, structure controllability, large surface areas, various surface modifications, high loading efficiency, biocompatibility and intrinsic biodegradability 114, 115. Given all these advantages, MOF can effectively incorporate some immunoagonists (e.g., CpG) 116 or Fe element 69 to boost antitumor immune response through the targeted modulation of TAMs into antineoplastic M1 type. Else, bismuth (Bi)-based nanomaterials have been applied to augment tumor radiotherapy efficacy 117, which also present good biocompatibility in clinic. On this basis, Qin et al. employed mesoporous upconversion nanophosphor (UCNP) doped with Bi to reprogramme the TAMs phenotype induced by X-ray radiotherapy for stimulating antitumor immune effects 70. In this system, upconversion luminescence was significantly enhanced due to the addition of Bi ion, which contributed to the increased radiosensitivity 118. This UCNP showed a mean diameter of 85 nm with a mesoporous structure based on their proper surface area of 22.96 m^2^/g and pore size of 3.8 nm, which further favored the physical loading of another chemotherapeutic doxorubicin (DOX) for combination immunotherapy.

### Natural carrier-based nanomaterials

At present, considerable evidences have proved that biomimetic natural nanocarriers derived from diverse biological membrane are emerged as immunotherapeutic systems for enhanced antitumor immunity. Among of them is natural killer (NK) cell membranes, which can play a role of immune-inducer in inherently promoting tumoricidal pro-inflammatory M1-type polarization and activating adaptive immune response for cancer immunotherapy 72, 119. Deng et al. used NK cell membranes coated photosensitizer-loaded NPs for synergistic photodynamic-immunotherapy 72, which improved the immunotherapeutic efficacy of NK cell membranes and ultimately achieved anticipated antitumor potency both in eliminating primary tumors and inhibiting metastatic tumors. Another one reported was bacterial outer membrane vesicles (OMVs) that were produced from gram-negative bacterial membranes 120, which could act as immunomodulatory delivery vehicles for TAMs-focused cancer therapy. OMVs possess most of the immunogenic components from their parent bacteria, which facilitate their immunostimulatory effect on modulating and stimulating the adaptive immune response for cancer immunotherapy 121. Chen et al. applied bioinspired OMVs coated chemotherapeutic micelles for boosting antitumor immunotherapeutic effect and preventing tumor metastasis 71, in which OMVs elicited antitumor immune response by modulating the immunoactivity of different immune cells such as TAMs, and antitumour micellar NPs also played immunomodulatory roles in improving tumoricidal activity of CTLs. In addition, M1 macrophages membrane-based nanovesicles that can specifically target M2-TAMs have been used as an immunoregulator to facilitate the repolarization of immunosuppressive M2-TAMs for boosting antitumor immune response 74. Also, red blood cell (RBC) membranes have been selected to modify drug delivery based on NPs due to their easy isolation, biocompatibility and various modifications 122, 123, which can avoid clearance by blood circulation and the RES and extend the half-life of drug. Han and colleagues developed bioinspired NPs based on immunostimulatory agents-encapsulated PLGA NPs coated with galactose-modified RBC membrane for actively targeting TAMs and reversing TAMs phenotype 73, which obviously remodeled the immunosuppressive TME and augmented tumor immunotherapy.

Cell membranes-derived exosomes (also called as small extracellular vesicles) can also be used as nanomaterials for immunotherapy. Dario et al. have indicated that these exosomes can be significantly uptaken by macrophages, which means that this class of nanocarrier has a certain potential for TAMs targeting 124. Hence, the application of exosomes as nanocarriers of TAMs reprogramming molecules can effectively repolarize an immunostimulatory M1-phenotype. Additionally, Fan et al. constructed a donor cell-derived exosome carrier system modified with anti-PD-L1 and anti-CD40 antibodies to perform combination tumor immunotherapy, where dual antibodies not only served as targeting ligands but also as checkpoint blockade drugs for improving the therapeutic effect 125. Of note, such natural carriers have the characteristics of good biocompatibility, immune evasion, and high stability, and they can enable the enhanced immunotherapy by reversing checkpoint-mediated immune suppression. Inspired by this idea, researchers can consider to employ M2-type TAMs as the donor cells of exosomes and modify checkpoint blocking antibody on their surfaces, thus realizing the co-delivery of TAM-targeting drugs and the combined ICB immunotherapy.

In addition to biological membranes as natural vectors, it has been verified that the ink ejected from cuttlefish, composed of melanin, polysaccharides, oligopeptides, metals, etc. 126, can be utilized as natural immunomodulatory NPs to modulate macrophage-mediated immune responses through increase of M1 subpopulations and the improved recruitment of CTLs into tumor tissues, resulting in significant inhibition of tumor growth and metastasis 127. Considering physicochemical properties, these NPs from cuttlefish ink, featured with high dispersion and good biocompatibility, displayed a spherical morphology and a mean size of about 167 nm, which enabled safe and effective antitumor immunotherapy. Besides, Aljabali et al. have reviewed that through surface engineering of their capsids, virus-derived nanomaterials facilitate various potential applications for selective drug delivery in multiple therapies such as vaccine production and immunotherapy 128.

### Diverse functional modifications for TAMs-associated immune-nanotherapeutics

Although a variety of nanomaterials are available for TAMs-centered immunotherapeutic strategies, there are still severe challenges in the effective delivery of immunomodulatory drugs to target tissues and cells using nanoplatforms due to inherent *in vivo* biological barriers 87. To this end, functional modification of the nanoplatform is essential to obtain a smart weapon for boosting TAMs-focused anticancer immunotherapy. In this section, a brief summary of targeting, stimulus-response and TAM imaging in TAMs-relevant immune-nanotherapeutics is severally described.

### Targeting modification

As is well known, the aforementioned NPs possess the ability of passive targeted delivery resulted from the enhanced permeability and retention (EPR) effect, which enables the increased tumor accumulation of NPs on account of leaky tumor vasculature 129. Yet, EPR-dominated passive targeting capacity is restricted to the considerable differences between patients and tumor types in tumor vascularization and interstitial fluid pressure 16, which directly affects nanotherapeutic efficacy in clinical cancer treatment. More notably, the passive accumulation of NPs in tumor tissues does not mean their successfully delivery to the anticipated target cells thanks to the complex TME. For these reasons, passive targeted NPs carrying immunomodulatory agents also cannot easily access and regulate TAMs to stimulate antitumor immune response. Conversely, Wilhelm et al. indicated that active targeted delivery that relies on ligand-receptor interactions holds more advantages than passive targeted delivery through analysis of NPs delivery to tumors 130. Accordingly, reasonable incorporation of TAMs-targeting ligands onto the surface of the nanoplatform may not only significantly increase drug concentrations inside the TAMs via ligand-receptor affinity mediated endocytosis, but also further boost specific antitumor immunotherapeutic efficacy with decreased systemic side effects.

During the past decades, great efforts have been devoted to actively targeting M2-like TAMs using above-mentioned nanoplatforms surface-modified with diverse macrophage-affinity ligands. In general, **s**pecific TAMs-targeting ligands for the surface modification of nanocarriers are mainly classified as follows: first, for carbohydrates-associated ligands, both mannose 42, 50, 63, 78 and glucomannan (*Bletilla Striata* polysaccharide (BSP)) 53, 131 show unique affinity for mannose receptor (CD206) overexpressed on M2-like TAMs, which can facilitate the selective delivery of immunoregulators to TAMs. Moreover, galactose can also serve as a specific binding moiety to endow immune-nanomedicines the ability to actively target TAMs 55, 97, which is closely associated with high levels of macrophage galactose-type lectin (Mgl) from M2-like TAMs; second, for peptides-based ligands, current studies have identified that M2pep (a M2-macrophage binding peptide) and α-peptide (a scavenger receptor B type 1 (SR-B1) targeting peptide), that specifically and preferentially target M2-like TAMs, are often utilized as active targeting ligand to covalently grafted onto the surface of lipids NPs 41, micelles 46, polymeric NPs 51, and AuNPs 66; third, acid-relevant ligands, including sialic acid (SA) 132 and folate (FA) 44, have been used as effective targeting moieties of liposomal NPs to enhance drug delivery to TAMs for cancer immunotherapy, resulting from individually binding with Siglecs (SA-binding immunoglobulin-type lectins) and FA receptor β overexpressed on M2-like TAMs. Besides, given the specific expression of molecular marker proteins on M2-macrophage surface, relevant antibodies against macrophages may be designed as specific ligands for TAMs-targeting modification of nanocarriers 76. For example, Chen et al. used anti-PD-L1 nanobody against PD-L1 overexpressed on M2-TAMs in combination with mannose to targetedly modify liposomal NPs 133, which enabled the preferential delivery of therapeutic agents to TAMs.

### Stimulus-responsive modification

To ensure the therapeutic efficacy of immune-stimulating drugs after targeting the tumor immune microenvironment, stimulus-responsive groups can be reasonably introduced into NPs to trigger drug release in a spatiotemporal controlled manner, which is conducive to further promoting specific immune activation to enhance the efficacy of immunotherapy. Accordingly, in TAMs-centered immunotherapeutic strategies, such drug delivery nanoplatform should be intelligently designed to respond to common environmental stimuli mainly including internal response (acidic pH, glutathione (GSH), protease) and external response (heat and light), thereby facilitating drug delivery and timely exerting immunotherapeutic effect of the released drug within the targeted sites of TME.

Among internal stimuli, acidic pH is a commonly used responsive condition, which mainly involves in weak acid in TME and low-pH within endosome/lysosome. Generally, tumor acidic microenvironment-responsive NPs, that consisted of pH-sensitive material such as O-carboxymethyl-chitosan 42, PHA 53 and modified poly(β-aminoester)s 92, can rapidly disassemble to release most of drugs once they deposit in tumor site. For low pH response within tumor cells, some researchers used PLGA NPs loaded with ammonium bicarbonate (NH_4_HCO_3_) to respond to endosome/lysosome acidification (pH ~5-6), which could generate abundant CO_2_ and NH_3_ to facilitate the disassociation of NPs and disruption of endosome/lysosome membrane, thus effectively released phototherapeutic agents to cytoplasm of TAMs 50. For another study, researchers adopted pH-sensitive ZnO NPs as immunomodulatory nanocarriers 134, 135, which exhibited excellent controlled ability to promote DOX release by being dissolved at low pH 110. GSH, as another typical stimulus, is also extensively applied for redox-responsive drug release of nanoplatforms in exposure to enriched GSH in tumors. Given the high redox conditions inside the M2-like macrophages, disulfide bonds (-SS-) may usually be introduced into the NPs, thereby achieving the specific delivery and release of drugs 63. Furthermore, redox/pH dual responses are often synchronously modified into nanocarriers, where rapid release of drugs can be first triggered by acidic pH in TME or endosome/lysosome and then by the high level of GSH in cytoplasm of tumor cells or TAMs 55, 61. Besides, it has been demonstrated that, MnO_2_ is capable of inevitable degradation in acidic TME with sufficient H_2_O_2_ 136, 137. Given this, a dual-responsive combination of pH/H_2_O_2_ based on MnO_2_ NPs have been used for on-demand drug release in TAMs-associated cancer immunotherapy 67, 83. Proteases, like matrix metalloprotease 2 (MMP2) presented in TME, can be considered as a response for controlled release of nanomedicines. MMP2-based functional peptide sequence is generally introduced into NPs for MMP2 response. For example, PEG-MMP2 peptide sequence-modified liposomal system and PLGA NPs have been developed for responding to MMP2 in TME, resulting in the detachment of long PEG chains and synchronously facilitating the exposure of targeted ligand 44, 52.

For external stimuli, hyperthermia can be utilized as a physical stimulus for antitumor therapeutic response of NPs. There was a study about magnetic hyperthermia, namely that a liposome loaded with superparamagnetic NPs and CSF1R inhibitor BLZ945 were constructed for antitumor immune response under magnetic hyperthermia responses 138. Photo, identified as a common physical responsiveness, are usually applied for some inorganic NPs with optical/electronic properties, including AuNRs/NPs, Fe_3_O_4_ NPs, BP NPs, etc. As expected, these photo-responsive nanomaterials can achieve specific drug release in a spatiotemporal manner when exposed to specific light wavelengths 100.

### TAM imaging

The imaging of TAMs, particularly the M2-type TAMs is very important for guiding tumor therapy and assessing therapeutic efficacy. In clinic, it can help extract prognostic data, delineate tumor boundaries, direct tumor biopsies, etc. 139. Imaging macrophages can be effectively achieved by using biocompatible nanomaterials based on magnetic resonance imaging (MRI) or second region near-infrared (NIR-II) fluorescence imaging. For TAM MRI-imaging, MRI can easily detect the tissue distribution of NPs internalized by macrophages in both benign and malignant nodules, and thus feedback whether the corresponding tissues have malignant lesions. Fe_3_O_4_ nanomaterials have great application prospects for MRI imaging of macrophages 140. The assessment of Fe_3_O_4_ NPs to predict patient treatment response by imaging TAMs using MRI technique is currently in clinical trials and is believed to be put into clinical practice in the near future 91. In a study using NIR-II imaging by Zhu et al., they proposed a dynamic and highly sensitive approach for M2-TAMs imaging 141. To be specific, an Er-based rare-earth NIR-IIb nanoprobes modified with M2pep polypeptide were rationally designed for targeted imaging of M2-TAMs in glioblastoma, which enabled *in vivo* imaging under 980 nm laser excitation and was verified that their targeted imaging capacity could be achieved by their up-conversion fluorescence (540 nm) and downshifting fluorescence (1525 nm). From this study, such NPs have the potential to be used as a biocompatible diagnostic material for monitoring the tumor progression and assessing prognosis. In addition, He et al. developed a precise and deep-tissue multi-scale imaging technology with a surface-enhanced Raman scattering (SERS)/NIR-II optical AuDAg_2_S nanoprobe, which integrated SERS imaging and NIR-II in-depth biological fluorescence imaging, and eventually could achieve multidimensional tumor imaging in levels of single cell 142. Inspired by this study, researchers have to consider using such imaging technique in combination with TAM-targeting modification to image M2-type TAMs for directing TAMs-centered cancer immunotherapy.

## Current functional nanomedicines for different TAMs-targeting immunotherapeutic mechanisms

As previously mentioned, an ideal nanomedicine for TAMs-focused cancer immunotherapy is mainly composed of suitable nanomaterials with specific functional modifications, and targeted therapeutics encapsulated. In addition, TAMs play a key role in immune regulation by directly or indirectly promoting tumor cells to evade immune supervision. Moreover, considerable studies have revealed TAMs can trigger immunotherapy resistance, including checkpoint inhibitor therapy, adoptive cell transfusion, and tumor vaccines therapy 11, implying that TAMs-centered therapeutic strategies can synergistically augment the effectiveness of cancer immunotherapy. In general, the included therapeutic agents in nanomaterials need to hold the ability to directly or indirectly impact on immunosuppressive M2-like TAMs, which is very beneficial for reshaping immunosuppressive TME and improving antitumor immunogenicity. Currently, the underlying mechanisms of TAMs-focused immune-nanomedicines mainly involve three different therapeutic strategies, including (i) blockage of bone-marrow-derived TAMs recruitment, (ii) depletion of tumor-resident TAMs population, and (iii) reprogramming of TAMs phenotype 143. Various TAMs-targeting molecular mechanisms based on functional nanomedicines in cancer immunotherapy are summarized in **Table 2**.

### Blockage of bone-marrow-derived TAMs recruitment

**Figure 4:**
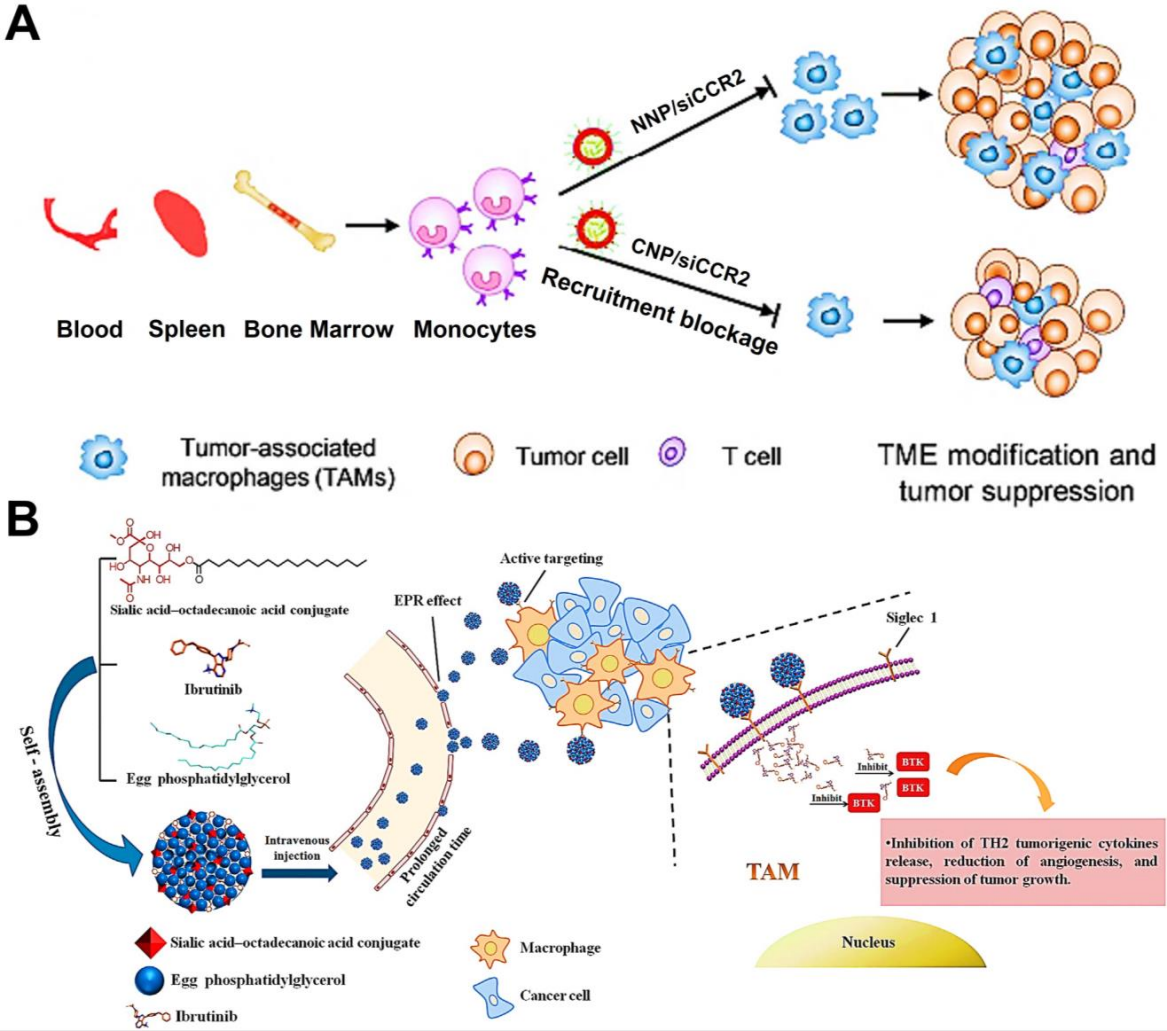
** (A)** CCR2 siRNA-encapsulated cationic nanoparticles (CNP/siCCR2) could more efficiently interrupt monocyte recruitment from peripheral blood to tumor tissues and decrease TAMs abundance, resulting in tumor microenvironment modification and tumor growth suppression. Adapted with permission from 79, copyright 2018 American Chemical Society. **(B)** Schematic illustration of SA/IBR/EPG nanocomplexes that increase tumor accumulation, inhibit BTK in TAMs, and exert immunotherapeutic effects. Adapted with permission from 77, copyright 2019 Elsevier Ltd. Abbreviations: siCCR2: CCR2 siRNA; CNP/siCCR2: siCCR2-encapsulated cationic nanoparticles; NNP/siCCR2: siCCR2-encapsulated neutral nanoparticles; TME: tumor microenvironment; BTK: Bruton's tyrosine kinase.

TAMs in tumor tissue are partially derived from circulating Ly6C^+^CCR2^+^ monocytes 149. To our best knowledge, the interaction between a tumor-derived chemokine CCL2 and monocytes-expressed chemokine receptor CCR2 can determine the recruitment of monocytes into tumor tissues, and this CCL2-CCR2 signaling axis is considered as a primary molecular regulator for monocyte recruitment in various tumors 150. Thus, it is feasible to impair the CCL2-CCR2 signaling axis to decline the generation of TAMs from bone-marrow-originated monocytes by targeting and impacting the monocytes. Some targeted therapeutic drugs such as small molecular inhibitors or antibodies, that can specifically target and suppress this signaling axis, have achieved superior therapeutic efficacy in preclinical and clinical trials 150, 151. In addition, CCR2 siRNA (siCCR2) can also be used as a promising targeted agent for interrupting monocyte recruitment. To efficiently deliver siCCR2 to monocytes, Shen et al. constructed siCCR2-loaded cationic PEG-PLA NPs for breast cancer therapy, and verified that these positively charged NPs could abundantly accumulate in monocytes (**Figure 4A**) 79. With these cationic NPs, siCCR2 could be efficiently delivered to monocytes and significantly inhibited CCR2 expression in monocytes, thereby alleviating the immunosuppressive degree of TME through blockage of bone-marrow-derived TAMs recruitment and improving antitumor and anti-metastasis potency.

It has been found that the generation of protumoral TAMs is closely associated with Bruton's tyrosine kinase (BTK) overexpressed in TAMs 152, which facilitates the recruitment of bone-marrow-cell infiltration into tumors, subsequently inducing the polarization of M2-like TAMs for fostering tumor growth via triggering immunosuppression 153. Considering this mechanism, the blockage of BTK can become a potential strategy that suppresses bone-marrow-originated TAMs recruitment for abolishing the TAMs-mediated immunosuppression and boosting antitumor immunity 154. In a research by Qiu et al. 77, they used ibrutinib (IBR, an irreversible BTK inhibitor) encapsulated in actively targeted SA-modified nanocomplexes as a potent TAMs-regulator for sarcoma immunotherapy (**Figure 4B**). These nanocomplexes possessed high loading capacity, increased half-life and small particle diameter (about 30 nm), which effectively delivered IBR to the tumor site and actively internalized by TAMs via SA-targeting. Then tumor immunosuppression caused by M2-TAMs was successfully blocked by inhibition of myeloid-cell recruitment responsible for IBR-induced BTK downregulation in TAMs, thus remarkably inhibiting tumor growth with negligible systemic toxicity.

### Depletion of tumor-resident TAMs population

Tissue-resident TAMs tend to polarize to an immune-deactivated M2 phenotype that can elicit tumor immune suppression in many malignancies 10, which have been shown to restrain the tumor-specific immune response by inducing the dysfunction of DCs and CTLs, along with releasing abundant growth factors, cytokines and proteases 155. Hence, current nanomedicines-based antitumor approaches aiming to reduce the population of M2-TAMs may be in favor of remodeling the immunosuppressive TME and improving anti-tumor immunity.

**Figure 5:**
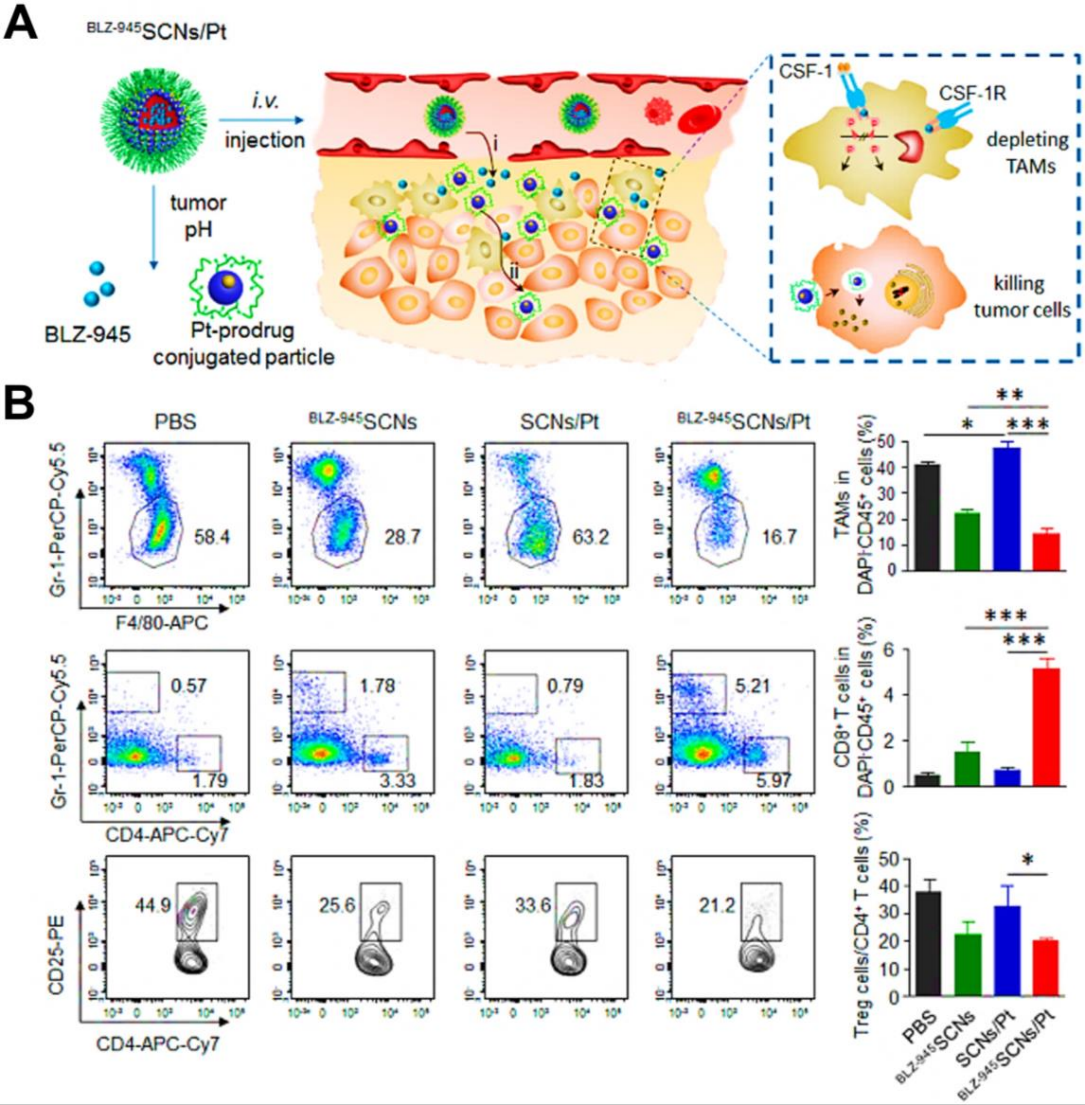
** (A)** Schematic illustration showing the mechanism of spatial delivery of BLZ-945 and Pt-prodrug to TAMs and tumor cells. **(B)** Relative abundance of various immune cells in 4T1 tumor tissues at the end of treatment by flow cytometry. These cells included CD45^+^CD11b^+^Gr1-F4/80^+^ TAMs, CD45^+^CD11b-CD8^+^ T cells and CD45^+^CD11b-CD4^+^ T cells, and Treg cells. Adapted with permission from 80, copyright 2017 American Chemical Society. Abbreviations: SCNs: sensitive cluster nanoparticles; Pt: platinum.

**Figure 6:**
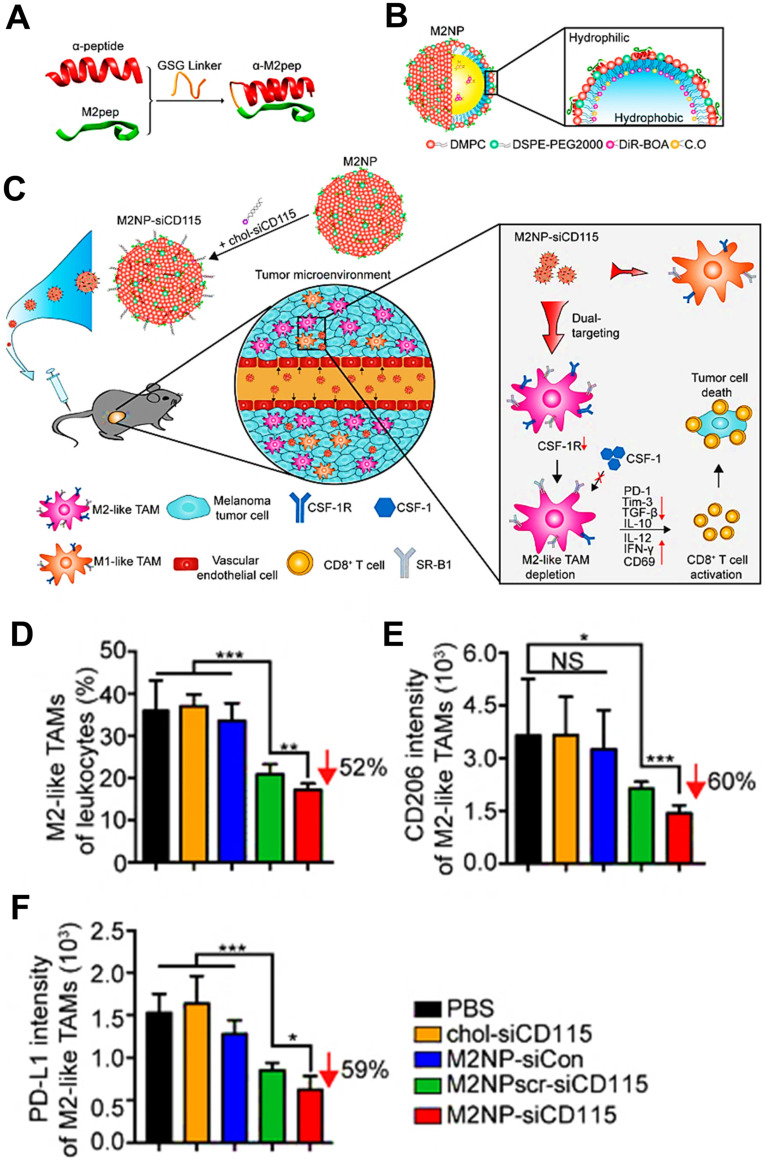
** (A)** Hybrid approach of the fusion peptide α-M2pep.** (B)** Structure and components of M2NP. **(C)** M2NP-based delivery of siRNA for CSF-1R silencing and immunoregulation via synergistic dual targeting of M2-like TAMs *in vivo*. **(D)** Proportion of M2-like TAMs among the total tumor infiltrating leukocytes in mice after the indicated treatment. **(E)** Flow cytometry data showing the CD206 expression by M2-like TAMs in tumors after the indicated treatment. **(F)** PD-L1 expression on M2-like TAMs after the indicated treatment. Adapted with permission from 41, copyright 2017 American Chemical Society. Abbreviations: α-peptide: a scavenger receptor B type 1 (SR-B1) targeting peptide; M2pep: M2 macrophage binding peptide; siCD115: anti-CSF-1R siRNA.

CSF1-CSF1R chemokine axis has been viewed as the crucial signaling pathway responsible for polarization of TAMs to immunosuppressive M2 phenotype 82. Direct inhibition of CSF1R in CSF1-CSF1R signaling axis by some molecular targeted inhibitors (e.g. BLZ-945, anti-CSF1R antibody and CSF1R siRNA) is capable of highly decreasing the abundance of TAMs in TME by inhibition of cell growth or survival, thereby eliciting CD8^+^ T cells-based immune response against tumor progression 156, 157. For instance, Shen et al. designed tumor acidic microenvironment-sensitive dendrimer NPs to co-load BLZ-945 via hydrophobic interactions and platinum (Pt) prodrug via covalent conjugation, which spatially targeted TAMs and tumor cells for cancer chemo-immunotherapy 80. From results, these NPs were able to disassemble to small particles in response to low pH of tumor site 158 and facilitated the quick release of BLZ-945, which was then preferentially uptook by TAMs to contribute to TAMs elimination by CSF1R blocking (**Figure 5A**). Meanwhile, small Pt prodrug particles were released to penetrate into deep tumor region for further tumor eradication. As shown in **Figure 5B**, this BLZ-945-loaded NP significantly reduced the population of TAMs (nearly 47.6%) in tumor tissues and obviously increased the abundance of activated CD8^+^ T cells along with sharp decrease of Treg cells in CD4^+^ T cells, indicating that inhibition of TAMs survival successfully enhanced the activation of CD8^+^ T cells-mediated immune response. In a recent report, Xie et al. introduced CSF1R blocker BLZ-945 into the bioactive nanovaccines, which effectively inhibited M2-like TAMs in tumor tissues through a series of pH/size/charge transitions, and finally remodeled the immunosuppressive TME for potentiating immunotherapeutic efficacy 159. Besides, Li et al. fabricated an alginate hydrogel loaded with pexidartinib-encapsulated NPs, which could effectively release small molecular pexidartinib to inhibit CSF1R for eliminating M2-TAMs 160. Depletion of TAMs further facilitated local and systemic delivery of anti-PD-1 antibody-conjugated platelets to suppress post-surgery tumor recurrence. In a study by Qian et al. 41, they used M2pep and α-peptide-modified lipid NPs to systemically transport anti-CSF1R siRNA (siCD115) to melanoma and selectively targeted M2-like TAMs for molecular-targeted cancer immunotherapy, thereby specifically blocking the survival signal of M2-like TAMs through inhibition of CSF1-CSF1R pathway by siCD115 and abundantly eliminating them from tumor tissues, followed by the effective activation of antitumor immune responses (**Figure 6A-C**). By analysis of tumor immune environment, it was found that dual-targeting lipid NPs carrying siCD115 remarkably decreased the number of TAMs population accounting for about 52% in tumor tissues, along with a 60% decrease of CD206 (M2-type marker) and a 59% decline of PD-L1 on M2-TAMs (**Figure 6D-F**). Moreover, these targeted nanomedicines resulted in an obvious inhibition of immunosuppressive IL-10 and TGF-β and a dramatic increase of immunostimulatory IL-12 and IFN-γ, and a 2.9-fold augment of CD8^+^ T cell infiltration in TME.

Several bisphosphonates, like clodronate (CLO) and alendronate (ALN), have been formulated into appropriate immunostimulatory nanomedicines for targeted depletion of M2-type macrophages. Sousa et al. used liposomal-encapsulated CLO to produce high cytotoxity toward macrophages for significant elimination of TAMs 40, which possibly resulted from their induced apoptosis via blocking mitochondrial adenine nucleotide translocase inside macrophages 161. Zhan et al. aimed to build a targeted immune-nanomedicine based on a conjugation of ALN with BSP (ALN-BSP) to efficiently target and specifically inhibit TAMs in TME for cancer immunotherapy 131. As presented in *in vivo* experiments, these ALN-containing targeted NPs preferentially accumulated in TAMs and effectively deleted TAMs by ALN-triggered apoptosis, eventually recovering local immune surveillance for suppressing tumor growth. In their follow-up study 52, they prepared MMP-2-responsive PLGA NPs to encapsulate ALN-BSP for TAMs-targeting cancer immunotherapy. As expected, these immune-nanomedicines could effectively accumulated in tumor site and released ALN-BSP to actively target TAMs due to MMP-mediated disassembly, thereby effectively suppressing tumor progression by remodeling tumor immune microenvironment.

**Figure 7:**
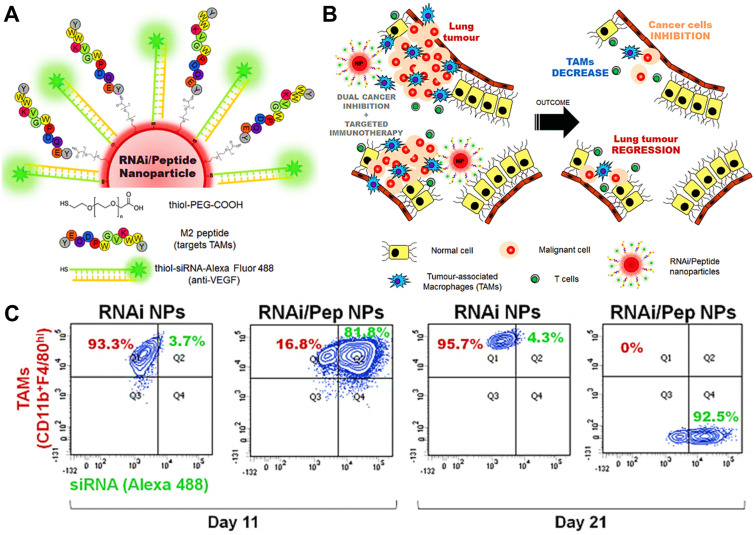
** Nanoparticle-based strategy to deliver RNAi for VEGF silencing in both TAMs and lung cancer cells. (A)** AuNPs functionalized with thiolated-PEG-COOH conjugated to TAMs-targeting peptide (M2pep) and thiolated anti-VEGF siRNA labeled with Alexa Fluor 488. **(B)** Schematic of the outcome of the proposed combined silencing therapy (immunotherapy targeting TAMs and cancer cells) *in vivo* via highly specific and potent NPs administered directly to bronchial airways. **(C)** CD11b F4/80hi expression on TAMs (CD11b^+^F4/80hi) for mice treated with RNAi- and RNAi-M2pep-AuNPs at days 11 and 21. Adapted with permission from 66, 2015 WILEY-VCH Verlag GmbH & Co. KGaA, Weinheim. Abbreviations: RNAi: RNA interference; siRNA: small interfering RNA; Pep: peptides.

The vascular endothelial growth factor (VEGF) highly expressed in M2 TAMs can promote tumor progression and metastasis 162, so the usage of siRNA against VEGF can become a promising approach for improving tumor suppression by interrupting the recruitment of inflammatory M2 TAMs. Conde et al. employed M2pep-modified AuNPs for targeted delivery of VEGF siRNA (siVEGF) to M2-like TAMs in lung tumor tissues 66, thereby significantly eradicating M2 population from the tumor site by actively silencing VEGF pathway and boosting a T cell-priming immune response that led to durable tumor inhibition (**Figure 7A, B**). *In vivo* outcomes during 21d tumor treatment showed that VEGF silencing by siVEGF-encapsulated M2pep-AuNPs observably decreased the number of macrophages in lung tissues, and resulted in an about 95% reduction of M2-like TAMs in tumor tissues (**Figure 7C**).

**Figure 8:**
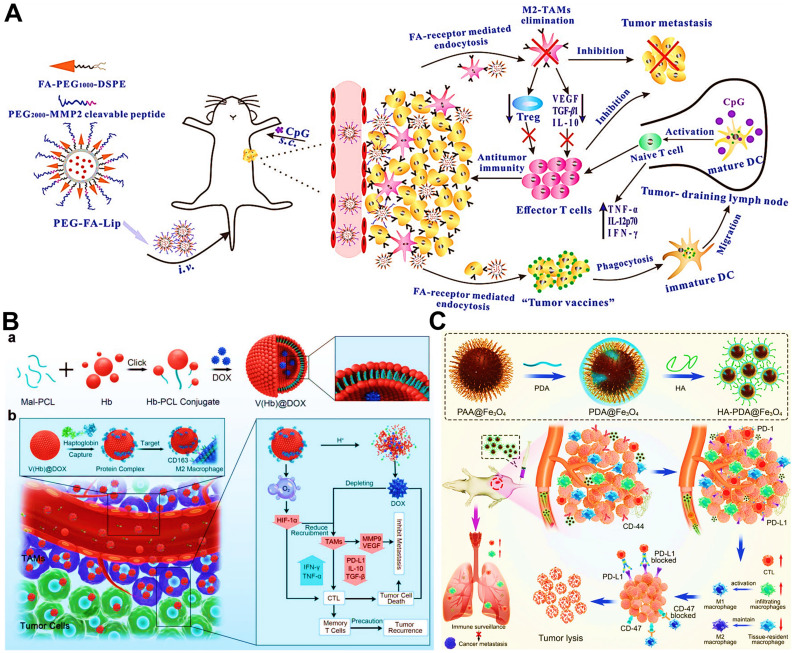
** (A)** The mechanism of antitumor immunity triggered by PEG-FA-Lip based targeting chemotherapy in combination with CpG immune adjuvant therapy. Adapted with permission from 44, copyright 2019 WILEY-VCH Verlag GmbH & Co. KGaA, Weinheim. **(B)** Schematic illustration of a) the design of the DOX-loaded RBC-biomimetic hollow nanovesicle (V(Hb)@DOX), and b) the V(Hb)@DOX-facilitated antitumor chemo-immunotherapy through TAM-targeting depletion and hypoxia alleviation. Adapted with permission from 81, copyright 2021 Wiley-VCH GmbH. (C) Schematic illustration of the preparation of HA-PDA@Fe_3_O_4_ NPs and HA-PDA@Fe_3_O_4_-synergized tumor immune therapy by the regulation of M1/M2 ratio and dual blockages of PD-L1/CD47. Adapted with permission from 164, copyright 2021 Wiley-VCH GmbH. Abbreviations: FA: folate; MMP 2: matrix metalloprotease 2; Lip: liposome; i.v.: intravenous; s.c.: subcutaneous; Mal-PCL: maleimide-functionalized PCL; Hb-PCL: hemoglobin-poly(ε-caprolactone); PAA: polyacrylic acid; HA: hyaluronic acid; PDA: polydopamine.

In addition to above mechanisms, it has been reported that doxorubicin chloride (DOX·HCl) can effectively kill TAMs 163. Inspired by this mechanism, Deng et al. developed M2-targeted/MMP2-responsive liposomes loaded with DOX for TAMs-associated immunotherapy against breast cancer (**Figure 8A**) 44. Of which, FA-modified liposome could specifically deliver DOX to both breast cancer cells and M2-like TAMs for inducing tumor immunogenic cell death and simultaneous depletion of M2-TAMs, which synergistically activated effector T cells immune response. Likewise, Wang et al. designed a chemotherapeutic DOX-loaded RBC-bioinspired hollow nanovesicle composed of hemoglobin (Hb) and PCL for TAMs-targeted cancer immunotherapy (**Figure 8B**) 81. In this system, DOX-encapsulated RBC-biomimetic NPs could actively target M2 TAMs by the affinity of Hb-haptoglobin (Hp)-CD163, and adequately deplete the cells. Concurrently, Hb-promoted O_2_ release also decreased M2-macrophages recruitment by alleviating tumor hypoxia. Finally, both strategies synergistically augmented CTLs-based antitumor immunity and significantly suppressed tumor metastasis and recurrence. To fulfill the depletion of M2 macrophages within tumors, Sun and his colleagues reported HA-PDA@Fe_3_O_4_ NPs were developed for remodeling the immune “cold” of TME through regulation of the ratio of M1/M2 subpopulations (**Figure 8C**) 164. With the chemotactic effect of HA, lymphocytes were first recruited to the tumor site and then polarized into M1-type stimulated by HA-PDA@Fe_3_O_4_ NPs, followed by mass production of different chemokines that could transformed tumor immune suppression through cascading amplification effects. More specifically, these HA-modified magnetite NPs simultaneously decreased relatively the populations of tumor-resident M2-TAMs by maintaining M2 macrophages, thus synergistically potentiating TAMs-focused tumor immunotherapy.

### Reprogramming of TAMs phenotype

**Figure 9:**
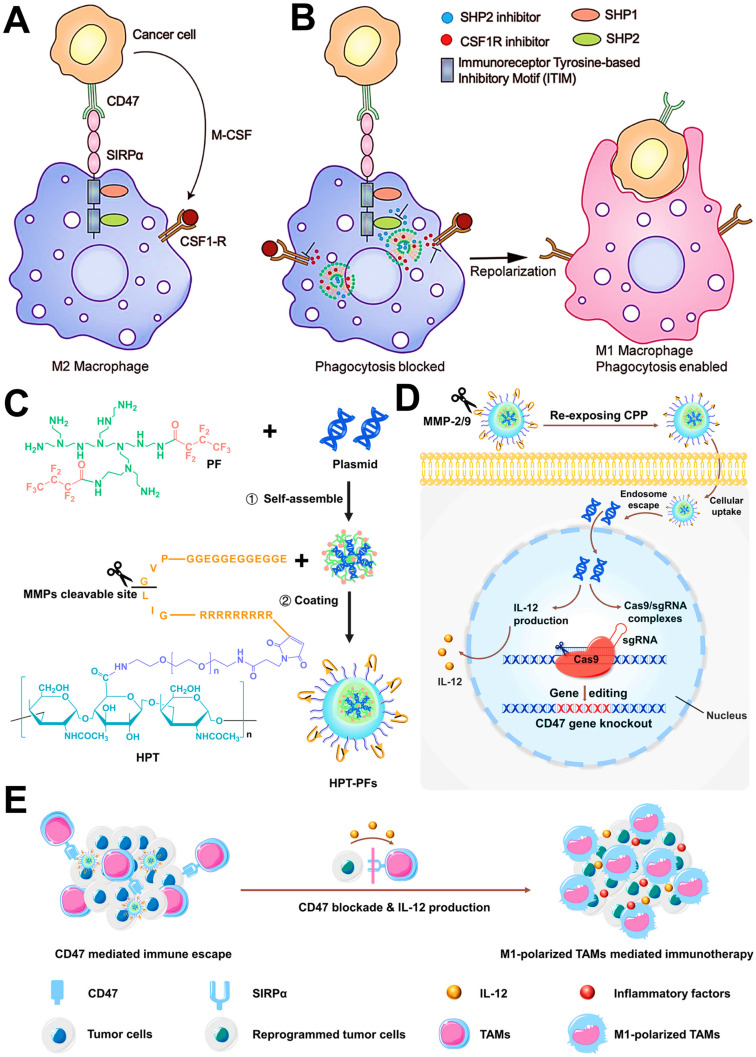
** (A)** Schematics show that simultaneous activation of the CSF1R pathway and the CD47-SIRPα pathway by cancer cells resulting in M2 polarized protumorigenic macrophages. **(B)** Schematics show deterministic co-delivery of DNTs to the M2 polarized macrophage leads to concurrent inhibition of CSF1R and SHP2, which results in repolarization of macrophages to an antitumorigenic M1 phenotype while simultaneously increasing the phagocytic index. Adapted with permission from 82, copyright 2019 WILEY-VCH Verlag GmbH & Co. KGaA, Weinheim. **(C)** Preparation of HPT-PF NPs loading with Cas9/sgRNA and pIL-12 plasmids. **(D)** Schematic illustration of MMP2/9 response and CPP-mediated tumor targeting of HPT-PF NPs, and IL-12 production and Cas9/sgRNA complexes-induced CD47 knockout by the transfection of Cas9/sgRNA and pIL-12 plasmids in melanoma cells. **(E)** Schematics show that the combination of CD47 blockade with IL-12 production synergistically promotes the M1-polarized TAMs for enhanced phagocytosis and secretion of inflammatory factors to elicit TAM-mediated immunotherapy. Adapted with permission from 57, copyright 2021 Elsevier Ltd. Abbreviations: MCSF: macrophage colony stimulating factor; CSF1-R: colony stimulating factor 1 receptor; SIRPα: signal regulatory protein α; SHP: Src homology region 2 (SH2) domain-phosphatase; PF: fluorinated polyethylenimine; HPT: hyaluronic acid-polyethylene glycol-tumor microenvironment sensitive peptides; CPP: cell-penetrating peptides; MMP-2/9: matrix metalloproteinases-2/9.

Repolarizing M2-subtype using immune-nanomedicines is also a widely used strategy for TAMs-focused cancer immunotherapy nowadays. Accumulating studies have attested the M2 reprogramming is related to the regulation of different signaling pathways by nanomaterials carrying molecular targeted agents. For instance, TAMs can promote the evasion of tumor cells from immune surveillance through both typical mechanisms of MCSF-CSF1R and CD47-SIRPα. It has been reported that dual blockages of the CD47-SIRPα and MCSF-CSF1R signaling axes by liposomes-encapsulated SHP2 and CSF1R inhibitors show great potential for TAMs-based cancer immunotherapy, which resulted in sufficient re-education of M2-TAMs to an active M1 subpopulation (**Figure 9A, B**) 82. Similarly, Kulkarni et al. adopted both CSF1R- and SIRPα-blocking antibodies to self-assembly form supramolecular NPs for concurrently disabling dual signaling axes, followed by the enhancement of M1 polarization and obvious therapeutic efficacies against melanoma and breast cancer 165. Chen et al. employed CaCO_3_ NPs loaded with anti-CD47 antibody for polarization of TAMs to M1-type through targeted inhibition of CD47-SIRPα pathway, thereby enhancing phagocytosis of cancer cells by M1-TAMs and initiating T cell-mediated antitumor immune responses 86. In addition to small molecular inhibitors or antibodies, one study by Lin et al. reported a new strategy of nanocarriers with CRISPR-Cas technology for TAM-targeting immunotherapy (**Figure 9C-E**), namely that hybrid polymeric NPs, composed of fluorinated-PEI (PF), HA and TME-sensitive peptides (TMSP), could highly effectively deliver Cas9/sgRNA and pIL-12 plasmids for promoting M1-polarized TAMs by the blockade of CD47 and IL-12-mediated immune-activation in melanoma 57.

**Figure 10:**
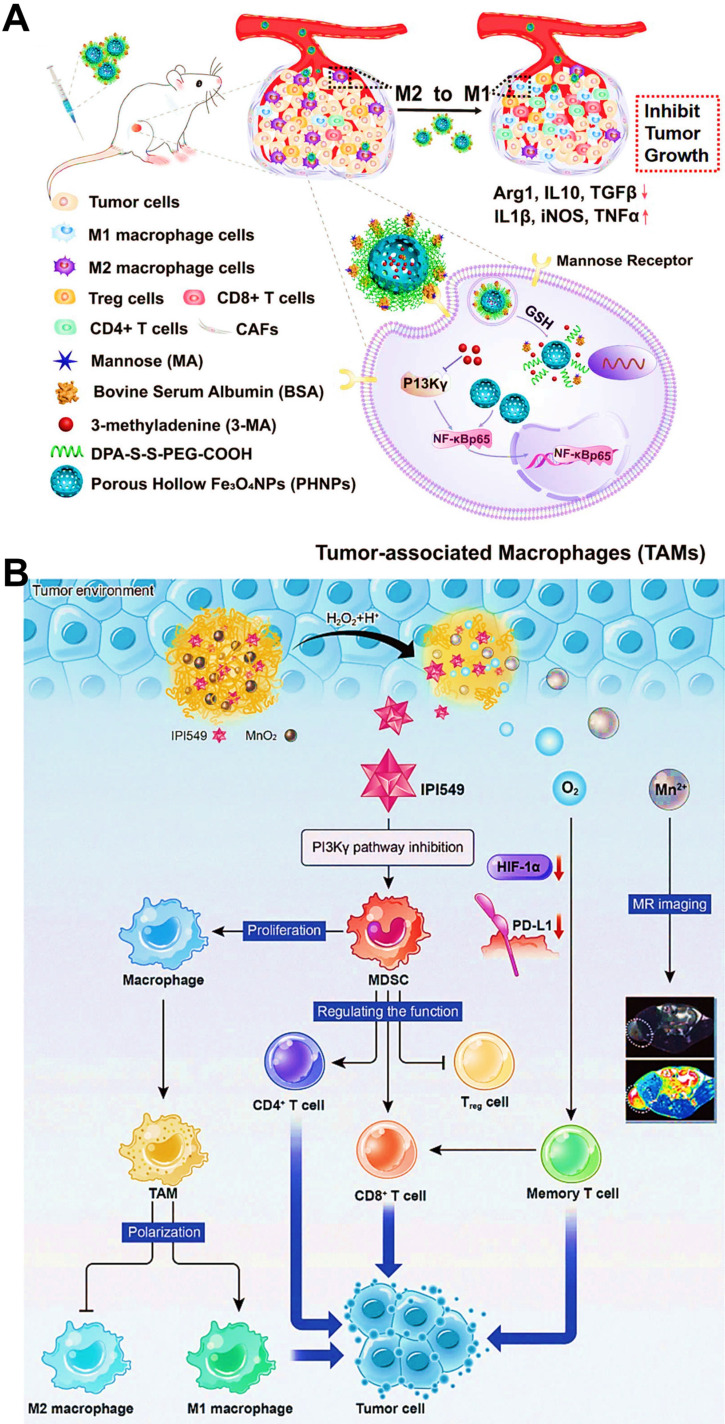
** (A)** PHNPs and 3-MA re-polarize TAMs to the M1-type through activating the protein of NF-κB p65, then remodelling the immunosuppressive microenvironment, thus activating immune response and inhibiting tumor growth. Adapted with permission from 63, copyright 2020 Royal Society of Chemistry. **(B)** Schematic illustration of BSA-MnO_2_-IPI549 nanoregulator as a stimulation-responsive platform to reshape tumor immune microenvironment for MR-guided immunotherapy. Adapted with permission from 83, copyright 2019 WILEY-VCH Verlag GmbH & Co. KGaA, Weinheim. Abbreviations: MDSC: myeloid-derived suppressor cell; MR: magnetic resonance.

The gamma isoform of phosphoinositide 3-kinase (PI3K-γ) highly expressed in myeloid cells (MDSCs or macrophages) holds a central switch between immune response and suppression, and regulates macrophages programming 166. Therefore, selection blockage of PI3K-γ pathway may suppress the infiltration of MDSCs into tumor sites, thus reshaping tumor immune microenvironment by transforming TAMs phenotype from the immunosuppressive M2-type to the pro-inflammatory M1-type. Li et al. developed M2-targeted mixed nanomicelles to deliver a PI3K-γ inhibitor (NVP-BEZ 235) and anti-CSF1R siRNA for the synergetic enhancement of TAMs repolarization and antitumor immune responses 46. In another study, Li et al. designed Fe_3_O_4_ NPs-based nanomedicines for the modulation of PI3K-γ-induced NF-κB p65 inactivation in M2-like macrophages (**Figure 10A**) 63. They incorporated a specific PI3K-γ inhibitor (3-methyladenine (3-MA)) into this M2-targeted/GSH-responsive nanoplatform, which further effectively upregulated the expression of NF-κB p65 by selectively blocking PI3K-γ pathway, and thus facilitated repolarization of TAMs into immunostimulatory M1-type for alleviating the immunosuppressive TME and augmenting antitumor immunity. Other than TAMs-related PI3K-γ mechanism, Yu et al. introduced a small molecular inhibitor IPI549 to BSA nanosystems containing MnO_2_ NPs for specific suppression of PI3K-γ pathway on MDSCs 83, which contributed to efficient polarization of antitumor M1-macrophage, accompanied by increased population of CTLs and declined population of Treg cells for boosting cancer immunotherapy (**Figure 10B**).

**Figure 11:**
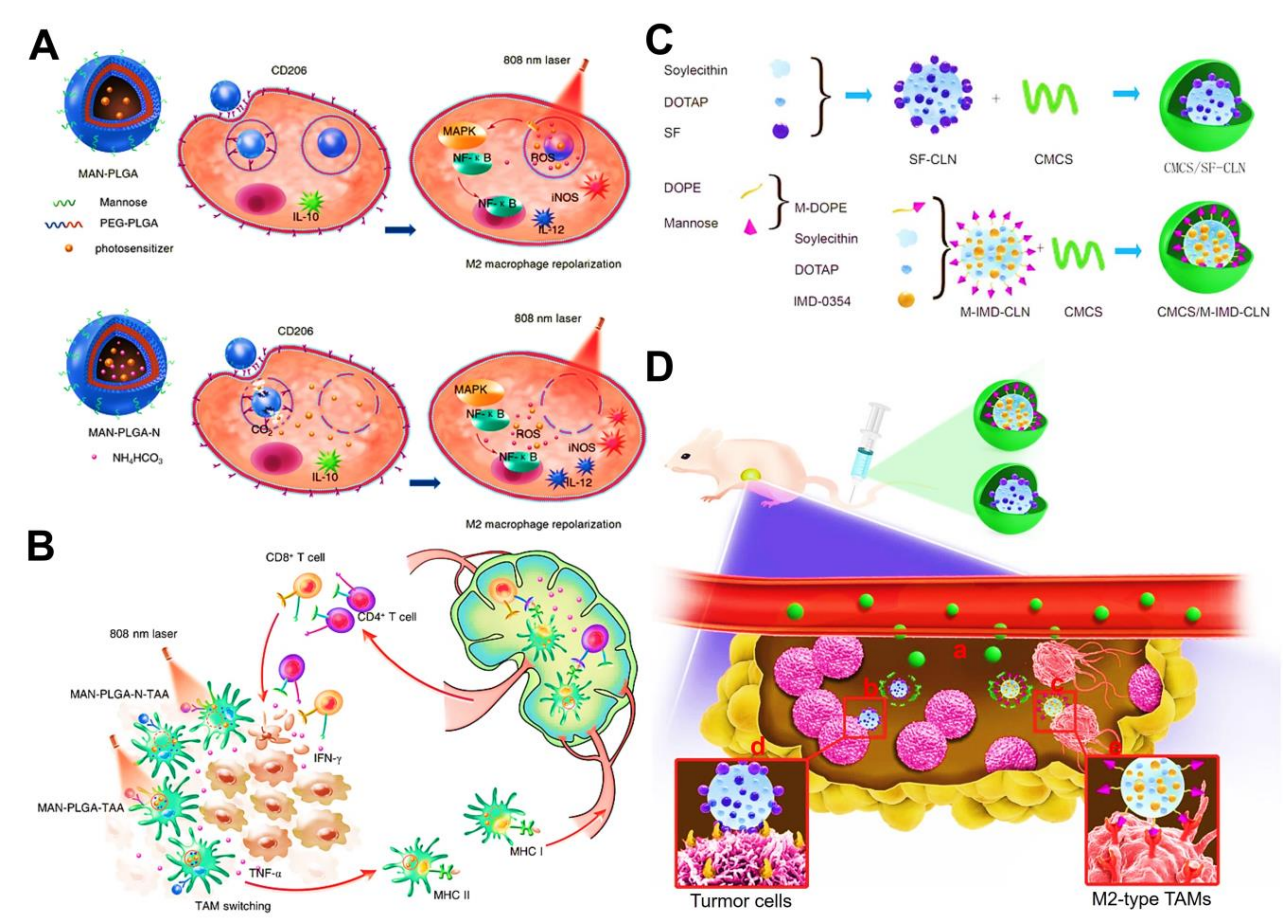
** (A)** Schematic illustrating the composition/structure of MAN-PLGA and MAN-PLGA-N nanoparticles and their mechanisms of activation of the M1 signal transduction and repolarization of M2 macrophages to M1 phenotype. **(B)** Schematic of TAM-directed cancer immunotherapy with nanoparticle-based ROS photogeneration. Adapted with permission from 50, copyright 2018 American Chemical Society. **(C)** Formulation of tumor cell targeting CMCS/SF-CLN and M2-type TAM targeting CMCS/M-IMD-CLN. **(D)** Co-administration of CMCS/SF-CLN and CMCS/M-IMD-CLN. a) CMCS/SF-CLN and CMCS/M-IMD-CLN can synchronous accumulate in tumor tissue. b) and c) Separated cells targeting ability due to the pH-responsive detachment of CMCS and exposure of different targeting ligands modified CLN. d) and e) Tumor cells targeted delivery by SF-CLN and M2-type TAM targeted delivery by M-IMD-CLN, respectively. Adapted with permission from 42, copyright 2019 Royal Society of Chemistry. Abbreviations: MAN: mannose; TAA: tumor-associated antigen; SF: sorafenib; CMCS: O-carboxymethyl-chitosan; CLN: cationic lipid-based nanoparticles.

As previously reported, the activation of NF-κB pathway may regulate the transformation of M2-to-M1 phenotype via some critical mechanisms in macrophages 167. For example, Shi et al. applied M2-targeted photodynamic PLGA NPs that could specifically trigger ROS generation within TAMs to effectively promote the polarization and antigen presentation of immune-activated M1-TAMs for enhanced specific antitumor immune activity 50, which was mainly ascribed to the activation of signaling pathways of mitogen activated protein kinase (MAPK) and NF-κB via the oxidation of cysteine residues of proteins 93 (**Figure 11A, B**). However, some studies proposed the opposite view that inactivating NF-κB pathway could dominate the reprogramming of M2-like TAMs 168. Especially, IKKβ, as a key regulator in NF-κB activation, has been considered as a potential therapeutic target for effective repolarization of M2-TAMs 168. Inspired by this view, Wang et al. used M2-targeted/pH-responsive lipid NPs encapsulating IMD-0354 (a IKKβ inhibitor) for selectively triggering M2-type TAMs repolarization through the inhibition of IKKβ, synergized with the chemotherapy of lipid NPs-formulated sorafenib (SF) for remodeling tumor immunosuppression and enhancing therapeutic potency (**Figure 11C, D**) 42. Besides, IKKβ, acts as a kinase that activates interferon regulatory factor 5 (IRF5), can facilitate the re-education of immunosuppressive M2 phenotype. Zhang et al. reported that M2-targeted polymeric NPs carrying IRF5/IKKβ-encoding mRNAs were employed to reverse the tumor-supporting state of TAMs in multiple tumor models, which further enhanced antitumor immunity and tumor suppression 169. In another study, Romulo S. et al. developed a methotrexate-loaded PLGA NP for reversing TAMs phenotype by inhibiting STAT3/NF-κB pathway in M2 macrophages, which was further combined with a PD-L1 antibody to amplify immunotherapeutic effect of breast cancer 145.

Other than NF-κB signaling-based mechanisms, some evidences have also testified that the M1-type polarization may be associated with expression regulation of the signal transducer and activator of transcription (STAT) 170. Ye et al. reported that chlorogenic acid (CHA) was served as an effective immunoregulator and then formulated into a M2-targeted liposomal nanomedicine for active delivery of CHA to TAMs in anti-glioblastoma immunotherapy 78. The CHA-based nanomedicines completely exerted their potentiated antitumor immunoactivity by adopting TAMs-focused immunotherapeutic strategy based on sufficient induction of M1 subtype via activation of STAT1 and suppression of STAT6. Moreover, Shobaki et al. fabricated a siRNA-loaded pH-sensitive cationic lipid NP for M2 re-polarization by silencing STAT3 and hypoxia inducible factor 1 α (HIF-1α) in TAMs, which also led to the reversion of tumor immunosuppression 146.

**Figure 12:**
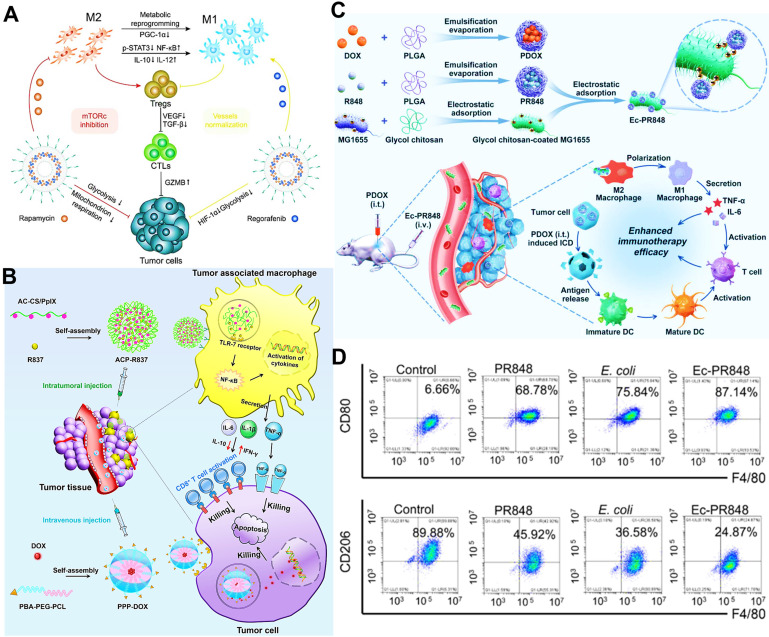
** (A)** A dual-targeting delivery liposomal system was designed with dual-modification of PD-L1 nanobody and mannose ligands for co-delivering an mTOR inhibitor (rapamycin) and an anti-angiogenic drug (regorafenib). The liposomes were able to target both TAMs and cancer cells that overexpressed PD-L1 and mannose receptors, and then efficiently reduced glycolysis, repolarized TAMs, inhibited angiogenesis, reprogrammed immune cells, and consequently arrested tumor growth. Adapted with permission from 133, copyright 2020 Elsevier Ltd.** (B)** Schematic illustration of the enhanced cancer chemo-immunotherapy resulting from intratumorally and intravenously injected nanomedicines. Of which, the immunostimulatory micelle ACP-R837 activated TAMs and promoted the secretion of cytokines, leading to an augmented antitumor immune response. Adapted with permission from 96, copyright 2019 Elsevier Ltd. **(C)** Schematic illustration of the formation of R848-loaded PLGA NP coated with *E. coli* (Ec-PR848) and TAMs re-polarization-promoted cancer immunotherapy. **(D)** Flow cytometric analysis of the proportion of M1 macrophages (F4/80^+^CD80^+^) and M2 macrophages (F4/80^+^CD206^+^). Adapted with permission from 148, copyright 2021 American Chemical Society. Abbreviations: mTOR: mammalian target of rapamycin; GZMB: granzyme B; TLR-7: toll-like receptor 7; i.t.: intratumoral; i.v.: intravenous.

mTOR signaling, a typical cellular metabolic pathway, plays an essential regulation in M2 macrophage polarization, mainly resulting from up-regulating the expression of p-STAT3 and IL-10 171. So, repolarizing TAMs by mTOR blockage may hold a great promise in anticancer immunotherapy. Chen et al. used rapamycin as an mTOR inhibitor and formulated it into TAMs-targeted liposomal nanomedicines for immunostimulatory M1 polarization via inhibition of mTOR signaling (**Figure 12A**) 133. Moreover, another anti-angiogenic regorafenib loaded by liposomes was concurrently applied to increase M1 population via vessels normalization. Dual-drug treatment using targeted liposomes successfully induced TAMs reprogramming to regulate CTLs-priming antitumor immune responses, along with increased immune-promoting factors and decreased immune-suppressive factors.

Activation of TLRs-mediated signaling in macrophages by using some agonists, including CpG 65, 95, imiquimod (R837) 85, 96 and resiquimod (R848) 75, 172, is a conventional therapeutic strategy for TAMs re-education and their antitumor immune-activity recovery. For example, Shu et al. separately fabricated multifunctional biomimetic NPs or PLGA NPs loading with baicalin and a TLR9 agonist (CpG) for effectively repolarizing M2-like TAMs into tumoricidal M1 phenotype, which further reversed the tumor immunosuppressive state and augmented tumor-specific immune stimulation in anti-melanoma therapy 51, 73. In our previous research (**Figure 12B**) 96, we reported the immunostimulatory micellar platforms composed of protoporphyrin IX (PpIX)-grafted CS and immunoadjuvant R837 were designed to selectively deliver R837 to TAMs owing to the affinity between CS and CD206, and further activated TAMs through the interaction of R837 with TLR7 receptor on the lysosomal membrane of TAMs, along with abundant production of pro-inflammatory cytokines (TNF-α and IL-6) for eliciting CD8^+^ T cells-mediated tumor cytotoxicity. Meanwhile, another targeted micelles-formulated DOX directly exerted chemotherapeutic activity against tumor cells, which was synergized with R837-induced immunotherapy for enhanced breast cancer therapy. Furthermore, Wei et al. applied R848-loaded PLGA NPs coated with nonpathogenic bacterium *E.coli* to realize the re-polarization of M2-type macrophages (**Figure 12C**) 148. These biomimetic nano-agonists could be effectively delivered to hypoxic tumor and phagocytosed by M2 macrophages. On one hand, R848 released could activate TLR7/8 signaling for M1-type induction. On the other hand, the existence of lipopolysaccharide (LPS) and flagellin in *E.coli* could separately activate TLR4/5 related pathways for the conversion of M2 to M1 173, 174. Hence, the obvious increase of M1 and the significant decline of M2 were presented after incubation with these nano-agonists (**Figure 12D**).

Current studies have proved that miR may play an important role in regulating macrophage polarization and activation. Among most miRs, miR155 has been used as a modulator for effectively restraining the TAMs-associated cytokine production via targeting C/EBPβ 175 and repolarizing immunosuppressive M2 type into immune-activated M1 type *in vitro* 176, suggesting that increasing miR155 inside the TAMs holds a numerous promise in inducing M1 polarization and relieving tumor immune suppression for inhibiting tumor progression. Hence, Liu et al. used pH/GSH dual-stimuli sensitive and M2-targeted polypeptide nanocarriers for TAMs-targeted miR155 delivery 55. Consequently, administration of multifunctional NPs-encapsulated miR155 markedly upregulated miR155 content within TAMs, thereby facilitating the reprogramming of TAMs phenotype by regulating the ratio of M1/M2 population, and activating T and NK cells-priming immune responses for robust tumor regression. For another therapeutic miR, let-7b (a miR mimic) can act as a potent TLR-related agonist for regulating macrophages via specifically binding to TLR7 receptor and concurrently inhibiting immunoregulatory IL-10 secretion 177. Huang et al. constructed M2-targeted and pH-responsive polysaccharide nanocomplexes to fulfil the therapeutic delivery of let-7b for actively modulating TAMs repolarization and elevating immune-antitumor efficacy toward breast cancer 53.

According to previous researches, spleen tyrosine kinase (SYK) plays a key role in the induction of the macrophage-mediated immune response after macrophages engulf crystals or NPs 178, and endoplasmic reticulum stress can result in SYK activation, which may trigger immune response in immune cells 179. Inspired by these ideas, Su et al. constructed a polyaniline-based glyco structure coating Au NPs (Au@PG NPs) to induce macrophage polarization form M2 to M1 in lung cancer immunotherapy, which was dependent on NP size with smaller NPs performing better than larger one 180. The reason for this was that endocytosis-mediated cellular uptake of NPs induced size-dependent endoplasmic reticulum stress, which facilitated the activation of SYK, and thus led to immune regulations with TAM repolarization.

**Figure 13:**
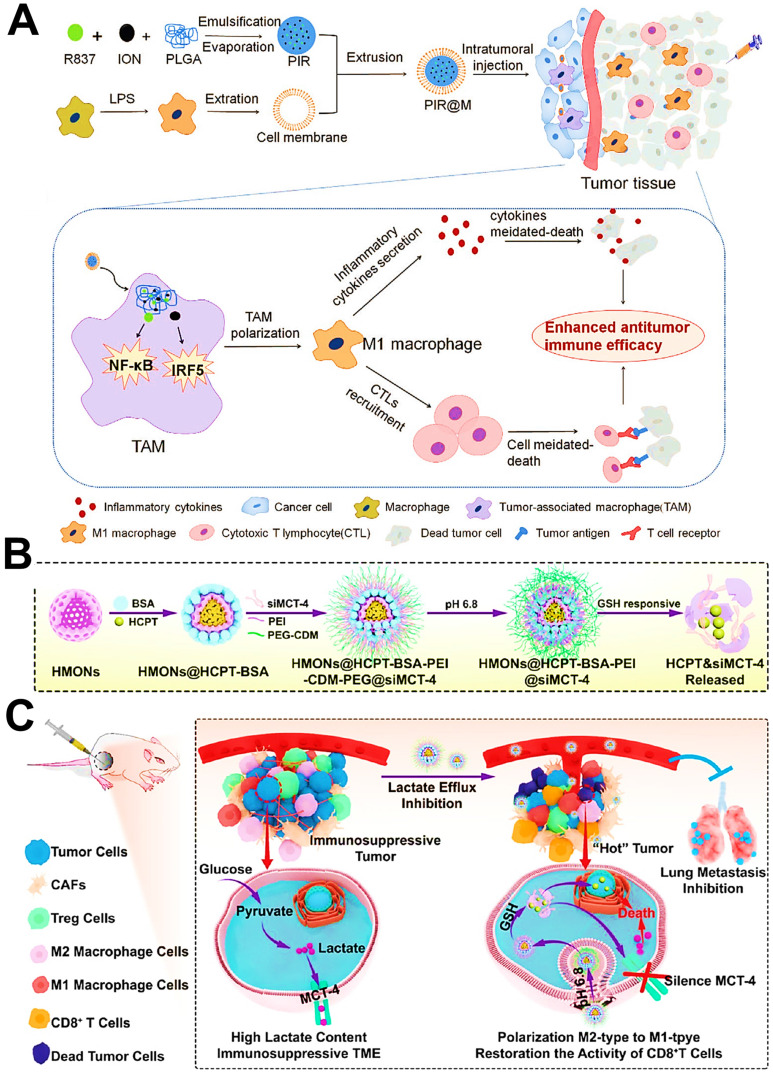
** (A)** Scheme of the preparation of M1 macrophage cell membrane-coated nanocarriers loaded with Fe_3_O_4_ NPs and R837 (PIR@M NPs). The PIR@M NPs polarized the TAMs to M1 phenotype through activing the NF-κB and IRF5 signaling pathways after intratumoral injection. Adapted with permission from 85, copyright 2020 Wiley-VCH GmbH. **(B)** Synthesis of HMONs@HCPT-BSA-PEI-CDM-PEG@siMCT-4 and stimuli-responsive degradation. **(C)** Schematic illustration of the cascaded responsive nanoplatform for enhancing tumor chemo-immunotherapy via inhibiting lactic acid efflux. The nanoplatform directly induces tumor cell apoptosis though HCPT and the increased intracellular lactate, then transforms immunosuppressive tumors to “hot” tumors, polarizes the TAM phenotype from M2 type to M1 type, and restores CD8^+^ T cell activity via inhibiting lactate efflux. Adapted with permission from 61, copyright 2020 American Chemical Society. Abbreviations: ION: iron oxide nanoparticle; LPS: lipopolysaccharide; HMONs: hollow mesoporous organosilica nanoparticles; BSA: bovine serum albumin; HCPT: 10-hydroxycamptothecine; MCT-4: monocarboxylate transporter 4.

Increasing evidences have demonstrated that FDA-approved Fe_3_O_4_ NPs 113, 181 or Fe^3+^-chelated NPs 56, 182, 183 may induce the phenotypic shift of TAMs from tumor-supportive M2 type to tumor-suppressive M1 type, and potentiate macrophage-modulating cancer immunotherapies. The M1-polarization mechanism may be attributed to the fact that Fe_3_O_4_ NPs mainly activate the IRF5 signaling via generated Fe^3+^ instead of the ROS-induced NF-κB signaling (**Figure 13A**) 85, or Fe_3_O_4_ NPs may stimulate inflammatory responses by initiating the TLR4 signaling on surface of macrophages 184. Furthermore, another mechanism study has pointed out that the adequate accumulation of Fe^3+^ inside the macrophages can promote the phenotypic switching of macrophages by activation of NF-κB signaling pathway 185. In other research of metal-based NPs, Zheng et al. used Cu_2-x_Se (CS NPs) to realize the repolarization of M1-like macrophages in melanoma 186. Concretely speaking, the production of ROS mediated by CS NPs in the macrophages could trigger auto-ubiquitination of tumor necrosis factor receptor-associated factor 6 (TRAF6), thus activating the interferon regulatory factor 5 (IRF5) and reprogramming the TAMs.

To the best knowledge of another reprogramming mechanism, aerobic glycolysis-produced lactate in the TME dominates phenotype conversion of M1-to-M2 TAMs and inactivation of CD8^+^ T cells 187, facilitating the avoidance of tumor cells from innate immune system. So, the regulation of lactate levels in the TME may become a powerful cancer immunotherapy strategy. It has been found that the monocarboxylate transporter 4 (MCT4) on surface of tumor cells guards the gate of lactate outflow 188, thus silencing MCT-4 protein by using siRNA is capable of increasing the tumor intracellular lactate and decreasing the extracellular lactate to reshape the TME 189. Li et al. employed smart organosilica-based nanoplatforms to deliver anti-MCT-4 siRNA (siMCT-4) for blocking tumor intracellular lactate efflux via MCT-4 silencing, resulting in the shift of TAMs phenotype from M2 to M1 and re-activation of CD8^+^ T cells (**Figure 13B, C**) 61. Simultaneously, the increased intracellular lactate and antineoplastic HCPT loaded by NPs synergistically promoted tumor regression and metastasis suppression. Altogether, this novel strategy successfully fulfilled the transformation of immune “cold” tumors to “hot” tumors, which can be more conducive to the arrest of tumor progression.

In addition to those three main strategies, a novel TAMs-focused immunotherapy approach, CAR-macrophage (CAR-M) therapy, has been reported recently. Chen et al. developed an injectable hydrogel co-loaded with macrophage-targeted genetic engineering nanoparticles (pCAR-NPs) and CD47 antibody for immunotherapy of GBM to prevent postoperative recurrence of GBM 190. On one hand, pCAR-NPs performed *in situ* gene editing of local macrophages in postoperative GBM lumen, to form CAR-M that could target the removal of glioma stem cells (GSCs). On the other hand, co-delivered CD47 antibody blocked the tumor "don't eat me" signal, synergistically enhancing the phagocytosis efficiency of CAR-M on GSCs, and facilitating its antigen presentation of CAR-M to activate the specific T cell immune response, which cleared the residual GSCs after surgery, formed immune memory, and avoided GBM recurrence. Overall, we believe that this novel immunotherapeutic strategy based on CAR-M should be paid more attention and actively studied by researchers, and we hope that more research reports on this topic will emerge in the future.

## Conclusions and perspectives

TAMs, as the major immune cell types in TME, usually interact with other immune cells or tumor cells to create a tumor immunosuppressive microenvironment. TAMs thereby can be seen as an appealing therapeutic target for combination cancer immunotherapy. Recent advances in TAMs-focused cancer immunotherapy with multifunctional nanomedicines have highlighted three common therapeutic strategies aimed at targeting TAMs: blockage of bone-marrow-derived TAMs recruitment, depletion of tumor-resident TAMs population, and reprogramming of TAMs phenotype. With the unique advantages of functional nanocarriers in tumor-targeted drug delivery, they can effectively accumulate at tumor sites, and then selectively deliver immunomodulatory reagents to TAMs or indirectly affect the functional regulation of TAMs, thus creating an effector T cells favorable environment, often accompanied by increased infiltration, activation, maturation, and proliferation of immune-effector cells (e.g., CTLs), and decreases in the number of immunosuppressive cells (e.g., Treg and MDSCs) and secretion of immunosuppressive factors.

Although these three TAMs-focused nano-immunotherapies have shown good application progress in preclinical basic research, they still have their own potential side effects or limitations. For blockage of myeloid cell recruitment, some targeted therapeutic agents are used to interfere with the entry of exogenous macrophages into the tumor site, which may lead to a relative reduction in the population of M2 TAMs. Nonetheless, its essence is that the naturally existing resident macrophages in TME have not been effectively inhibited or eliminated, which makes the cunning tumors continue to employ these resident TAMs to perform tumor progression by the expression of biological proteins, cytokines or chemokines. Likewise, if tumor-resident TAMs are eliminated only by targeted drugs without simultaneously preventing the recruitment of bone marrow cell-derived macrophages, which is still possible to lead to accumulation of M2 macrophages in TME, further resulting in tumor development. Therefore, the clever integration of these two strategies into a nanosystem may not only avoid the occurrence of the above problems, but also achieve better therapeutic effects.

In terms of the strategy of TAMs repolarization, whether bone marrow-derived or tumor-resident macrophages, the adoption of repolarization inducers (small molecule inhibitors, immune agonists, nucleic acid drugs or antibodies) can contribute to the polarization of macrophages from M2 to M1. However, some of these polarization inducers may lead to abundant M1 macrophages in other normal tissues besides tumors, resulting in unanticipated proinflammatory or even excessive immune responses, which reflects the potential side effects of this class of treatment. Altogether it is important to understand and overcome these issues in TAM-based tumor immunotherapy before entering clinical trials.

Additionally, for the development of pluripotent nanomedicines, their properties such as category, size, charge, shape, functional modification and optimal selection of therapeutic agents based on TAMs-associated molecular mechanisms need to be judiciously considered, which will enable increased solubility, improved pharmacokinetics, enhanced delivery stability, effective tumor accumulation, precise delivery and minimal systemic toxicity. In general, TAMs-targeted cancer immunotherapies usually use intelligent nanomedicines to fulfill preferential drug delivery to M2-like TAMs at tumor site. However, M2-type macrophages with specific receptor expression may be distributed in other normal tissues throughout the body, and some M2 phenotype receptors like mannose receptor and Siglecs are also overexpressed in DCs or other cell types, which significantly restricts the delivery efficacy of nanomedicines, and results in an inability to accurately target TAMs for stimulating potent antitumor immune response. The comprehension of complexity and heterogeneity of macrophage phenotype will facilitate the development of precise TAMs-targeting immunotherapeutic nanomedicines. Else, it has been reported that TAMs are surrounded by a dense network of extracellular matrix, which may severely impede the intratumoral delivery of nanomedicines and their accessibility to TAMs 191. Altogether, it is very necessary to rationally design nanomedicines to make them accurately target and effectively regulate TAMs for anticancer immunotherapy.

In clinic, the presence of unacceptable serious toxicity from immunotherapies, as well as the low response rate, are two troublesome problems to consider in the preclinical and clinical development of cancer immune nanomedicines 192. Moreover, cancer patients often show obvious individual differences in immune status, genetics, tumor type and microenvironment, which may further affect the outcome of cancer immunotherapy. Especially in terminal cancer patients, sufficient activation of the immune system by an immunotherapeutic strategy to elicit a powerful response is a slow and rough process in the actual clinical treatment 16. Thus, these common issues should be further improved to achieve maximal clinical efficacy with minimal side effects.

Hitherto, very few of these therapies has been approved for clinical use. Even though FDA-approved ferumoxytol have been evidenced with macrophage reprogramming ability to promote TAMs repolarization to M1 phenotypes for cancer therapy, and these findings have not yet been further translated into clinical trials. Furthermore, because of the complex preparation procedure, stability of manufacture process and product, and rigorous quality control, great efforts should be devoted in the translational research of macrophage-centred immune-type nanomedicines for separate or combinational cancer therapy. Clearly, extensive validation in preclinical research is still required for accelerating clinical translation of TAMs-focused anticancer immune-nanomedicines.

## Figures and Tables

**Table 1:** Various TAMs-focused functional nanomedicines for cancer immunotherapy

Nanomaterial	Component	Reagent	Drug-loading modality	TAMs-targeting ligand	Stimulus response	Cancer type	Refs.
Lipids/liposome	PC/DSPE-PEG2000	iCSF1R/DAF-2-DA	Hydrophobic forces	/	/	Breast cancer	39
	DSPG/Cholesterol	Zoledronate/Clodronate	Hydrophobic forces	/	/	Breast cancer	40
	DMPC/DSPE-PEG2000/Cholesterol oleate	CSF1R-siRNA (siCD115)	Insertion of lipid monolayer	α-peptide/M2pep	/	Melanoma	41
	Soya lecithin/DOTAP/DOPE	Sorafenib/IMD-0354	Hydrophobic forces	Mannose	pH	Liver cancer	42
	Soybean phospholipid/Cholesterol/DSPE-PEG2000	Regorafenib	Hydrophobic forces	Alanine-alanine-asparagine	/	Breast cancer	43
	DSPE-PEG	DOX	Hydrophobic forces	FA	MMP2	Breast cancer	44
	DSPC/Cholesterol/PEG-DMG	BisCCL2/5i mRNA	Electrostatic compression	/	/	Liver cancer	45
Polymeric micelle	PEI-stearic acid/DSPE-PEG	BEZ 235/CSF1R-siRNA	Hydrophobic forces/Electrostatic interaction	M2pep	/	Pancreatic cancer	46
	DOTAP/PEG-PCL/mPEG-PLA	MIP-3β plasmid	Electrostatic interaction	FA	/	Breast cancer	47
	PEG-P(L-Arg)	L-arginine	Covalent polymerization	/	/	Colorectal cancer	48
	PAMAM dendrimer	BLZ-945/Pt-prodrug	Hydrophobic forces/Covalent conjugation	/	pH	Breast cancer	49
Polymeric NP	PEG-PLGA	ICG/TiO_2_	Nucleation	Mannose	pH/Light	Breast cancer	50
	PLGA/PDA	Baicalin/Hgp peptide/CpG	Nucleation/Electrostatic adsorption	M2pep/α-peptide	/	Melanoma	51
	PEG-PLGA/ALN-BSP	ALN	Covalent conjugation	Glucomannan (BSP)	MMP2	Liver cancer	52
	PEG-PHA	Let-7b	Electrostatic interaction	BSP	pH	Breast cancer	53
	BSA	DSF-Cu/Regorafenib	Hydrophobic forces	Mannose/TfR-binding peptide T12	/	Glioma	54
	PLL-PLC/PEG-PLL	miR155	Electrostatic adsorption	Galactose	GSH/pH	Melanoma	55
	PEG-PDA	Fe^3+^	Chelation	/	Light	Colon and breast cancer	56
	PF/HA-PEG-TMSP	Cas9/sgRNA and pIL-12 plasmids	Electrostatic compression	/	MMP2/9	Melanoma	57
	HA-PEI	miR125b	Electrostatic bound	HA	/	Lung cancer	58
C-based NP	Gadofullerene (Gd@C82)/β-alanines	Anti-PD-L1 antibody	/	/	/	Breast cancer	59
	Graphene oxide/PEG/PEI	CpG	Electrostatic adsorption	/	Light	Colon cancer	60
Si-based NP	Organosilica/BSA/PEI/PEG	HCPT/siMCT-4	Hydrophobic forces/ Electrostatic adsorption	/	pH/GSH	Melanoma and breast cancer	61
P-based NP	Black phosphorus/PEGylated HA	/	/	/	Light	Breast cancer	62
Metal-based NP	Fe_3_O_4_/PEG/BSA	3-MA	Hydrophobic forces	Mannose	GSH	Breast cancer	63
	Fe_3_O_4_/PEG	OVA	Covalent conjugation	/	/	Melanoma	64
	AuNR/PEG	DOX/CpG	Electrostatic adsorption/Au-S coordination	/	Light	Liver cancer	65
	AuNP/PEG	VEGF siRNA	Au-S coordination	M2pep	/	Lung cancer	66
	MnO_2_/PEG	Ce6/DOX	Hydrophobic forces	/	pH	Breast cancer	67
	Al and Ru-based MOF	Anti-PD-1 antibody	/	/	Light	Colorectal cancer	68
	Fe-based MOF	Diclofenac	Hydrophobic forces	M2pep	/	Liver cancer	69
	Bi-based UCNP	DOX	Hydrophobic forces	/	/	Lung cancer	70
Natural carrier	Bacterial outer membrane vesicles/DSPE-PEG/F127	Tegafur	Hydrophobic forces	/	/	Melanoma	71
	NK cell membrane/mPEG-PLGA	TCPP	Nucleation	/	Light	Breast cancer	72
	Erythrocyte membrane/DSPE-PEG/PLGA	Baicalin/Hgp peptide/CpG	Nucleation/Electrostatic adsorption	Galactose	/	Melanoma	73
	M1 macrophage-derived exosome	Anti-PD-L1 antibody	/	/	/	Colon cancer	74
	Cancer cell membrane/PLGA	R848	Hydrophobic forces	M2pep	/	Melanoma	75

**Table 2:** Various TAMs-targeting molecular mechanisms based on functional nanomedicines in cancer immunotherapy

Strategy	Nanomaterial	TAMs-targeting reagent	Therapeutic mechanism	Cancer type	Refs.
Blockage of bone-marrow-derived TAMs recruitment	Polymeric NP	CCR2-siRNA	Blockage of TAMs recruitment by siCCR2 inhibiting CCR2 expression in monocytes and impairing CCL2-CCR2 pathway	Breast cancer	79
	Nanocomplex	IBR	Active internalization of IBR-loaded NPs by TAMs via SA targeting and inhibition of myeloid-cell recruitment responsible for IBR-induced BTK downregulation in TAMs	Sarcoma	77
Depletion of tumor-resident TAMs population	Polymeric NP	BLZ-945	TAMs elimination facilitated by BLZ-945-mediated CSF1R blocking	Breast and colon cancer	144
	Lipid NP	siCD115	Specifically block the survival signal of M2-like TAMs through inhibition of CSF1-CSF1R pathway by siCD115	Melanoma	41
	Liposome	CLO	Induction of TAMs apoptosis by blocking mitochondrial adenine nucleotide translocase inside macrophages via CLO	Breast cancer	40
	Polymeric NP	ALN	Effectively delete TAMs by ALN-triggered apoptosis	Sarcoma	131
	AuNP	VEGF-siRNA	Eradicate M2-TAMs population from the tumor site by siVEGF actively silencing VEGF pathway	Lung cancer	66
	Liposome	DOX	Eliminate M2-like TAMs through DOX-triggered apoptosis	Breast cancer	44
Reprogramming of TAMs phenotype	Liposome	SHP2 and CSF1R inhibitors	Re-education of M2-TAMs by dual blockages of the CD47-SIRPα and MCSF-CSF1R signaling axes	Breast cancer	82
	CaCO_3_ NP	Anti-CD47 antibody	Reprogramming of M2-TAMs through targeted inhibition of CD47-SIRPα pathway	Melanoma	86
	Polymeric NP	Cas9/sgRNA and pIL-12 plasmids	Promote M1-polarized TAMs by the blockade of CD47 and IL-12 production	Melanoma	57
	Lipid NP	BisCCL2/5i mRNA	Induce the polarization of M1-phenotype TAMs by neutralizing CCL2/5	Liver	45
	Mixed micelle	NVP-BEZ 235 and CSF1R-siRNA	Synergetic enhancement of TAMs repolarization by selection blockage of PI3K-γ and MCSF-CSF1R pathways	Pancreatic cancer	46
	Fe_3_O_4_ NP	3-MA	Upregulate the expression of NF-κB p65 by selectively blocking PI3K-γ pathway in M2-like macrophages	Breast cancer	63
	BSA NP	IPI549	Induction of M1-macrophage polarization via specific suppression of PI3K-γ pathway on MDSCs	Breast cancer	83
	PLGA NP	ICG and TiO_2_	Activation of MAPK and NF-κB pathways via the oxidation of cysteine residues of proteins by photo-triggered ROS	Breast cancer	50
	Lipid NP	IMD-0354	Inactivate NF-κB pathway through the inhibition of IKKβ	Liver cancer	42
	PLGA NP	Methotrexate	Blockage of STAT3/NF-κB signaling axis facilitates the conversion of M2-type to M1-type	Breast cancer	145
	Liposome	CHA	Sufficient induction of M1 subtype via CHA-triggered activation of STAT1 and suppression of STAT6 in TAMs	Glioblastoma	78
	Lipid NP	STAT3/HIF-1α siRNAs	M2 re-education via silencing STAT3 and HIF-1α within TAMs	Renal cell carcinoma	146
	Micelle	IKKβ-siRNA and AS1517499	Induce M2-to-M1 repolarization via silencing IKKβ and inhibiting STAT6 in TAMs	Breast cancer	147
	Liposome	Rapamycin	Repolarize TAMs by the blockage of mTOR signaling	Colon cancer	133
	PLGA NP	Baicalin and CpG	Repolarize M2-like TAMs by activating TLR9	Melanoma	51
	Polysaccharide NP	R837	Activate M1-TAMs through the interaction of R837 with TLR7 receptor on the lysosomal membrane of TAMs	Breast cancer	96
	PLGA-loaded *E.coli*	R848	R848-driven TLR7/8 signaling and *E.coli-*activated TLR4/5 pathways	Breast cancer	148
	Polypeptide NP	miR155	Upregulate miR155 level within TAMs	Melanoma	55
	Polymeric NP	let-7b	Specifically bind to TLR7 receptor and inhibit IL-10 secretion	Breast cancer	53
	Fe_3_O_4_ NP	/	Fe_3_O_4_ NPs activate the IRF5 signaling via generated Fe^3+^	Breast cancer	85
	Organosilica NP	MCT-4 siRNA	Block tumor intracellular lactate efflux via MCT-4 silencing	Melanoma	61

## Abbreviations

TAMs: tumor associated macrophages
ICB: immune checkpoint blockade
CAR: chimeric antigen receptor
CTLs: cytotoxic T lymphocytes
TME: tumor microenvironment
SiO_2_: mesoporous silica
Fe_3_O_4_: iron oxide
IL-12: interleukin-12
CXCL9/10: C-X-C motif ligand 9/10
IL-6: interleukin-6
PGE_2_: prostaglandin E_2_
IDO1/2: indoleamine-pyrrole 2,3-dioxygenase 1/2
COX-1/2: cyclooxygenase 1/2
PD-L1/2: programmed death ligand 1/2
Tregs: regulatory T cells
DCs: dendritic cells
MDSCs: myeloid-derived suppressor cells
CCL2/5: C-C motif ligand 2/5
MCSF: macrophage colony stimulating factor
CSF1R: colony stimulating factor 1 receptor
SIRPα: signal regulatory protein α
SHP-1/2: Src homology region 2 domain-phosphatases 1/2
NPs: nanoparticles
siRNA: small interfering RNA
iCSF1R: CSF1R inhibiting
NO: nitric oxide
PC: phosphatidylcholine
DSPE-PEG2000: 1,2-distearoyl-sn-glycero-3-phosphoethanolamine-N-(amino(polyethyleneglycol)-2000)
DMPC: 1,2-dimyristoyl-sn-glycero-3-phosphocholine
PEG: Poly(ethylene glycol)
PCL: poly(ε-caprolactone)
PLA: poly(lactide)
PEI: polyethyleneimin
iNOS: inducible NO synthase
L-Arg: L-arginine
CS: chondroitin sulfate
PAMAM: poly(amidoamine)
PLGA: poly(lactic-co-glycolic acid)
PDA: polydopamine
ICG: indocyanine green
TiO_2_: titanium dioxide
ROS: reactive oxygen species
BSA: bovine serum albumin
TLR: Toll-like receptor
ODN: oligodeoxynucleotide
PHA: PEG-histamine-modified alginate
PLL-PLC: n-butylamine-poly(L-lysine)-b-poly(L-cysteine)
miR155: MicroRNA-155
C: carbon
Si: silicon
P: phosphorus
GO: graphene oxide
HMONs: hollow mesoporous organosilica NPs
HCPT: 10-hydroxycamptothecin
MCT: monocarboxylate transporter
BP: Black phosphorus
HA: hyaluronic acid
AuNRs/AuNPs: gold nanorods/NPs
MnO_2_: manganese dioxide
ZnO: zinc oxide
CaCO_3_: calcium carbonate
FDA: Food and Drug Administration
MOF: metal-organic framework
UCNP: upconversion nanophosphor
DOX: doxorubicin
NK: natural killer
OMVs: outer membrane vesicles
RBC: red blood cell
RES: reticuloendothelial system
EPR: enhanced permeability and retention
BSP: *Bletilla Striata* polysaccharide
Mgl: macrophage galactose-type lectin
SR-B1: scavenger receptor B type 1
SA: sialic acid
FA: folate
Siglecs: SA-binding immunoglobulin-type lectins
GSH: glutathione
-SS-: disulfide bonds
MMP2: matrix metalloprotease 2
BTK: Bruton's tyrosine kinase
IBR: ibrutinib
Pt: platinum
CLO: clodronate
ALN: alendronate
VEGF: vascular endothelial growth factor
siVEGF: VEGF siRNA
DOX·HCl: doxorubicin chloride
Hb: hemoglobin
PI3K-γ: phosphoinositide 3-kinase
3-MA: 3-methyladenine
MAPK: mitogen activated protein kinase
SF: sorafenib
IRF5: interferon regulatory factor 5
STAT: signal transducer and activator of transcription
CHA: chlorogenic acid
HIF-1α: hypoxia inducible factor 1 α
R837: imiquimod
R848: resiquimod
PpIX: protoporphyrin IX
LPS: lipopolysaccharide
MCT4: monocarboxylate transporter 4
